# Distinct physiological, transcriptomic, and imaging characteristics of asthma-COPD overlap compared to asthma and COPD in smokers

**DOI:** 10.1016/j.ebiom.2024.105453

**Published:** 2024-11-23

**Authors:** Vrushali D. Fangal, Aabida Saferali, Peter J. Castaldi, Craig P. Hersh, Scott T. Weiss

**Affiliations:** a Channing Division of Network Medicine, Brigham and Women's Hospital, Harvard Medical School, Boston, MA, 02115, USA; b Department of Medicine, Harvard Medical School, Boston, MA, 02115, USA

**Keywords:** COPDGene, Asthma, COPD, Asthma-COPD overlap, Pulmonary function test (PFT), Quantitative computed tomography (QCT), Phenotypic characterisation

## Abstract

**Background:**

The clinical and pathological features of asthma and chronic obstructive pulmonary disease (COPD) can converge in smokers and elderly individuals as asthma-COPD overlap (ACO). This overlap challenges the diagnosis and treatment of the distinct aetiologies underlying these conditions.

**Methods:**

We analysed 2453 smokers (≥10 pack-years), aged 45–80 years, from the Genetic Epidemiology of COPD (COPDGene) Study, stratified as Control, Asthma, COPD, and ACO based on Global Initiative for Chronic Obstructive Lung Disease (GOLD) criteria. A comprehensive assessment was performed, encompassing symptomatology, pulmonary function tests (PFTs), complete blood counts (CBCs), bulk RNA sequencing (RNA-seq), and high-resolution quantitative computed tomography (QCT) imaging to evaluate clinical impact, lung function, systemic inflammation, and structural alterations contributing to disease progression across respiratory phenotypes. Differential expression (DE) analysis was performed using whole blood RNA-seq (BH-corrected FDR < 0.01), followed by Gene Ontology (GO) and Kyoto Encyclopedia of Genes and Genomes (KEGG) pathway enrichment analysis. Group differences were assessed using the Mann–Whitney U-test (MWU) or Chi-squared test ($\chi^{2}$), with Bonferroni correction applied for multiple comparisons. Multivariate linear regression models were used to adjust the associations between disease status and specific clinical outcomes for confounders, with one-way ANOVA and Tukey's Honest Significant Difference (HSD) post-hoc test applied for pairwise comparisons. Our analysis aimed to delineate the extent and variability of clinical features among disease phenotypes to guide targeted therapeutic strategies.

**Findings:**

Our study highlights distinct yet overlapping profiles across ACO, asthma, and COPD. We effectively isolated disease-specific mechanisms by comparing each phenotype to smoking controls (GOLD 0) while accounting for baseline smoking-related inflammation. ACO exhibited the most severe symptom burden, with significantly higher COPD Assessment Test (CAT) score (18.32, 95% CI: [17.02, 19.63], P < 0.0001) and Modified Medical Research Council (mMRC) Dyspnea score (2.14, 95% CI: [1.92, 2.35], P < 0.0001) compared to COPD and asthma. ACO also displayed reduced lung capacity (forced expiratory volume in 1 s [FEV_1_]: 52.5%, 95% CI: [50.08, 54.93], P < 0.0001) and airflow limitation (FEV_1_/forced vital capacity [FVC]: 0.55, 95% CI: [0.5471, 0.5546], P < 0.0001), closely resembling COPD but significantly worse than asthma. The inflammatory profile of ACO exhibited a mixed response, featuring elevated neutrophil counts (4.57 K/μL, 95% CI: [4.28, 4.86], P < 0.0001) and eosinophil levels (0.22 K/μL, 95% CI: [0.20, 0.25], P < 0.01), contrasting with the predominantly neutrophilic inflammation in COPD and the absence of systemic inflammation in asthma. Structurally, ACO demonstrated significant airway remodelling (Pi10: 2.87, 95% CI: [2.83, 2.91], P < 0.0001), intermediate emphysema (5.66%, 95% CI: [4.72, 6.60], P < 0.0001), and moderate small airway disease (parametric response mapping for functional small airway disease [PRM^fSAD^]: 22.94%, 95% CI: [21.53, 24.34], P < 0.0001), reflecting features of both asthma and COPD. COPD was characterised by more extensive emphysema (8.9%, 95% CI: [8.34, 9.45], P < 0.0001), small airway disease (PRM^fSAD^: 27.09%, 95% CI: [26.51, 27.66], P < 0.0001), and gas trapping (37.34%, 95% CI: [36.33, 38.35], P < 0.0001), alongside moderate airway remodelling. At a molecular level, DE analysis revealed enrichment of the Hypoxia-Inducible Factor 1 (HIF-1) pathway in ACO, highlighting unique hypoxia-driven metabolic adaptations, while COPD was associated with neutrophil extracellular trap (NET) formation and necroptosis. In contrast, asthma exhibited significant airway remodelling (Pi10: 2.09, 95% CI: [2.05, 2.13], P < 0.0001), minimal parenchymal damage, and no systemic gene expression changes.

**Interpretation:**

Collectively, our findings underscore the lung function impairments, systemic inflammation, molecular mechanisms, and structural correlates distinguishing ACO from COPD and asthma, emphasising the need for precise clinical management and the potential for novel therapeutic interventions.

**Funding:**

This work was supported by 10.13039/100000050National Heart, Lung, and Blood Institute (NHLBI) grants U01 HL089897 and U01 HL089856, as well as by 10.13039/100000002National Institutes of Health (NIH) contract 75N92023D00011. Additional support was provided by grants R01 HL166231 (C.P.H.) and K01 HL157613 (A.S.).

**Research in context**

**Evidence before this study**

Asthma and COPD are well-characterised respiratory diseases with distinct clinical, functional, and molecular features. The emergence of ACO challenges this binary classification, with critics contending that it blurs the boundaries between asthma and COPD rather than constituting a distinct entity. Distinguishing ACO is particularly difficult due to overlapping symptoms such as airflow limitation and exacerbations, heterogeneity in clinical presentation, varied inflammatory profiles, differences in disease progression, and the impact of smoking - all of which contribute to inconsistent diagnostic criteria and treatment strategies. Current guidelines offer limited clarity, and the exclusion of ACO patients from clinical trials further complicates the understanding of its unique features and underlying molecular mechanisms.

**Added value of this study**

Our study provides a refined understanding of ACO by positioning it on a continuum between asthma and COPD. Leveraging extensive data from smokers in the COPDGene cohort, we integrated symptomatology, exacerbation patterns, lung function (PFTs), blood inflammatory markers (CBCs), advanced morphological imaging (QCT), and whole blood transcriptomic data to define ACO as a hybrid intermediate phenotype on the asthma-COPD spectrum. ACO is shaped by the dynamic interplay of airway hyperreactivity, systemic inflammation, and structural lung remodelling, with contributions from both innate and adaptive immune responses. This distinguishes ACO from the more polarised profiles seen in asthma and COPD, where asthma is characterised by episodic airway obstruction, eosinophilic inflammation, and minimal parenchymal damage, and COPD is marked by irreversible airflow limitation, neutrophilic inflammation, emphysema, and gas trapping. The distinct enrichment of immune pathways—such as HIF-1-driven metabolic adaptations in ACO and NET formation in COPD—provides valuable insights into the underlying mechanisms, offering potential guidance for future phenotype-specific research and therapeutic interventions.

**Implications of all the available evidence**

Our study advances the understanding of ACO by positioning it as a distinct clinical and molecular entity, rather than merely an overlap of asthma and COPD. This perspective may prompt a reassessment of current diagnostic criteria to reflect the unique clinical and molecular features of ACO. Tailored treatment strategies that address the specific characteristics of ACO may enhance patient outcomes by providing more precise and individualised care. This evolving understanding has the potential to improve diagnostic accuracy and therapeutic efficacy beyond existing approaches primarily designed for asthma or COPD alone.

## Introduction

Asthma and COPD are the two most prevalent chronic respiratory diseases of the airways,^1^^,^^2^ each characterised by distinct clinical features and pathophysiological mechanisms. Asthma is primarily marked by episodic airway inflammation, reversible airway obstruction, and increased airway narrowing and mucus production.^3^ In contrast, COPD involves the presence of chronic bronchitis and emphysema, leading to irreversible airflow limitation.^4^ The coexistence of asthma and COPD traits within individual patients can give rise to a condition known as ACO, a syndrome that combines elements of both diseases.^5^

Diagnosis of these respiratory diseases tends to be more straightforward at the extremes of the age spectrum,^6^ with younger atopic individuals typically diagnosed with asthma, and older adults, particularly those with a history of smoking, more often diagnosed with COPD.^7^ The clinical overlap is primarily evident in either asthma patients with a history of tobacco smoking or non-smokers with long-standing asthma who progress to COPD later in life.^8^^,^^9^ Patients with ACO generally experience a poorer quality of life, evidenced by increased exacerbation frequency, more severe symptoms, accelerated decline in lung function, and consequently higher mortality rates and healthcare costs.^10^, ^11^, ^12^ Although ACO affects an estimated 11% of the population, its precise definition, grounded in molecular or pathological markers, remains insufficiently characterised.^13^^,^^14^ Elucidating the hallmark features of ACO, particularly those that distinguish the condition from asthma and COPD, is essential for the advancement of targeted therapeutic strategies.

Historically, the Global Initiative for Asthma (GINA) and the Global Initiative for Chronic Obstructive Lung Disease (GOLD) acknowledged ACO in a joint document, highlighting its heterogeneity and the shared inflammatory characteristics of asthma and COPD.^15^^,^^16^ However, this document was later retracted, reflecting the controversies in the understanding of ACO. The exclusion of ACO patients from asthma and COPD therapeutic trials has further hindered comprehensive understanding of this overlap syndrome.

The underlying inflammatory mechanisms, particularly at the molecular level, remain partially understood. Accumulating evidence has shown that T_H_2 cytokines like *IL-4*, *IL-5*, and *IL-13* associated with eosinophilic response, and T_H_1 cytokines like *IFN* γ, *IL-1*, and *IL-12* associated with neutrophilic response in the peripheral blood, have the potential to stimulate epithelial cells, smooth muscle cells, and fibroblasts contributing to airway hyperresponsiveness and remodelling.^17^, ^18^, ^19^ Advanced imaging techniques, such as QCT, have shown promise in identifying emphysematous changes and assessing small airway inflammation, offering potential insights into disease morphology.^20^ Given the overlapping clinical presentations and the shared inflammatory pathways of asthma and COPD, a comprehensive analysis encompassing clinical presentation, transcriptomic predispositions, and morphological characteristics is imperative. Such an approach is crucial for elucidating the distinct and convergent features of asthma, COPD, and ACO, thereby paving the way for targeted therapeutic strategies and improved patient outcomes.

Towards this goal, we conducted a multimodal assessment of the older smoking population in the COPDGene Study to elucidate the nuanced interplay between clinical manifestations and their underlying functional, molecular, and structural correlates, contributing to a refined understanding of these complex respiratory diseases.

## Methods

### COPDGene study

#### Population characteristics

The COPDGene Study is a multicentre, prospective, observational study that enrolled over 10,000 participants aged 45–80 years, with at least a 10 pack-year history of smoking, both with and without COPD, who self-reported their sex and identified as non-Hispanic White or African American. The study aims to characterise the full spectrum of smoking-related lung diseases, encompassing individuals with established COPD and those with a proclivity for COPD but without spirometric evidence. Additionally, it seeks to comprehensively define COPD phenotypes, particularly in cases with coexisting asthma or atypical spirometric patterns. Participants with other lung diseases (e.g., pulmonary fibrosis, extensive bronchiectasis), previous lung surgeries, active cancer, suspected lung cancer, or recent significant surgeries or hospitalisations were excluded to avoid confounding factors that could affect lung function or study outcomes. Pregnant women were excluded due to the risk posed by CT scans to the foetus. Our analysis focused on participants who completed both Phase 1 (2008–2011) and Phase 2 (2013–2016) visits. We excluded non-smokers, those with Preserved Ratio Impaired Spirometry (PRISm), and individuals meeting GOLD stage 1 criteria. Participants without chest CT data at either visit, those with reported changes in smoking status, or significant changes in lung volume were also excluded to ensure data consistency. COPDGene is registered under http://clinicaltrials.gov/ (Identifier: NCT00608764).

#### Classification criteria

We primarily used GOLD criteria based on post-bronchodilator spirometry measurements for classification. To ensure robustness, sensitivity analyses were conducted using the Lower Limit of Normal (LLN) criteria, as recommended by the American Thoracic Society (ATS)/European Respiratory Society (ERS) guidelines.^21^, ^22^, ^23^, ^24^ The LLN defines airflow limitation as an FEV_1_/FVC ratio below the 5th percentile of the predicted value, adjusted for age, sex, height, and ethnicity, corresponding to a Z-score of −1.645 in a healthy reference population.

### Participant data collection

#### Data collection

Each participant underwent pre- and post-bronchodilator spirometry using a standardised protocol with EasyOne™ Spirometer (ndd Medical Technologies, Zurich, Switzerland). Collected data included demographic details, medication use, medical history, responses to the modified ATS Respiratory Epidemiology Questionnaire^25^ and the St. George's Respiratory Questionnaire (SGRQ)^26^ (Full data collection forms are available on COPDGene website: www.COPDGene.org). Additional assessments included height, weight, blood pressure, and oxygen saturation (on room air). Symptom burden was measured using validated tools, including the CAT score^27^ and the mMRC Dyspnea Score.^28^ Peripheral whole blood samples were collected during the Phase 2 visit for CBC and RNA-seq analysis, indicative of each subject's current health status. Volumetric chest CT scans were obtained at full inspiration and end-expiration and analysed using Thirona lung quantification software.^29^ All data collection tools, including the ATS Questionnaire,^25^ SGRQ(26), RNA-seq,^30^ and Thirona lung quantification software,^31^ are validated and widely accepted.

#### Ethics

Participants were enrolled across 21 clinical study centres throughout the United States after obtaining approval from the Institutional Review Board (IRB) at each centre. Ethical approval for the study was granted by the respective IRBs, including the National Jewish IRB at National Jewish Health (HS-1883a); the Partners Human Research Committee at Brigham and Women's Hospital (2007-P-000554/2; BWH); the Institutional Review Board for Baylor College of Medicine and Affiliated Hospitals (H-22209, H-22202); Columbia University Medical Center IRB (IRB-AAAC9324); Duke University Health System IRB (Pro00004464); Johns Hopkins Medicine IRBs (NA_00011524); the John F. Wolf, MD Human Subjects Committee at Harbor-UCLA Medical Center (12756-01); Morehouse School of Medicine IRB (07-1029); Temple University Office for Human Subjects Protections IRB (11369); The University of Alabama at Birmingham IRB for Human Use (FO70712014); University of California, San Diego Human Research Protections Program (070876); University of Iowa Human Subjects Office (200710717); VA Ann Arbor Healthcare System IRB (PCC 2008-110732); University of Minnesota Research Subjects' Protection Programmes (0801M24949); University of Pittsburgh IRB (PRO07120059); UT Health Science Center San Antonio IRB (HSC20070644H); Health Partners Research Foundation IRB (07-127); University of Michigan IRBMED (HUM00014973); Minneapolis VAMC IRB (4128-A); and the Saint Vincent Hospital—Fallon Clinic—Fallon Community Health Plan IRB (1143). Written informed consent was obtained from each participant prior to inclusion, ensuring compliance with ethical standards for human research.

### Clinical variables

#### Symptom assessment

Symptom burden was evaluated using the CAT and mMRC Dyspnea scores. CAT score is an eight-item measure that assesses the impact of respiratory symptoms, including cough, phlegm, chest tightness, and breathlessness, on health status. Responses to each item range from 0 (no impact) to 5 (maximum impact), resulting in a total score between 0 and 40. The mMRC Dyspnea Score, a 5-point scale, was employed to subjectively measure breathlessness during physical activity, with higher scores indicating more severe breathlessness. The presence of chronic bronchitis (calculated) was evaluated using composite cough/phlegm questionnaire data. Emphysema prevalence was determined by the presence of emphysematous changes on quantitative CT scans over the past five years. Incidences of shortness of breath attacks (observed) and wheezing (observed in past year) were calculated based on patient-reported outcomes and medical records. Exacerbations (calculated) were categorised as ‘frequent’ if subjects reported two or more exacerbations (requiring steroids and/or antibiotics) in the previous year and ‘severe’ if they required emergency room visits or hospitalisation based on patient medical records and self-reported hospitalisation data.

#### Lung function assessment

Respiratory function was evaluated using a spectrum of PFTs, providing insights into lung mechanics and gas exchange, including percent predicted FEV_1_, Total lung capacity (TLC), Diffusing Capacity of the Lung for Carbon Monoxide (DL_CO_), as well as FEV_1_/FVC and resting arterial oxygen saturation (SaO_2_) (%). FEV_1_ represents the volume of air exhaled in the first second after a bronchodilator is administered, while FEV_1_/FVC ratio is indicative of the degree of airway obstruction. TLC, which reflects the maximum volume of air the lungs can hold, was measured from inspiratory chest CT scans and expressed as a percentage of predicted plethysmographic TLC. DL_CO_ assesses the efficiency of gas transfer from the alveoli to the blood in the pulmonary capillaries, while resting SaO_2_ denotes the percentage of oxygen-saturated haemoglobin at rest, measured via pulse oximetry.

#### Leukocyte assessment

Leukocyte counts were assessed using CBC and expressed in thousands per microlitre (K/μL), including total white blood cell (WBC) counts and differential counts for neutrophils, eosinophils, monocytes, and lymphocytes.

#### Quantitative lung imaging assessment

Emphysematous destruction in lung tissue was assessed by measuring the percentage of lung voxels with low attenuation, exhibiting densities below −950 Hounsfield Units (HU). Gas trapping was evaluated by identifying voxels less than −856 HU, representing the proportion of lung area retaining air post-expiration and indicating obstructive airway disease. Parenchymal lung density, measured as the 15th percentile lung density (HU), served as an index to assess the severity of emphysema. Pi10, calculated as the square root of the wall area of a hypothetical bronchus with an internal perimeter of 10 mm, was used to estimate airway wall thickness and thus gauge airway remodelling. PRM^fSAD^ quantified the percentage of lung affected by small airways disease by mapping areas of functional decline on CT scans. Airway wall thickening (wall area %) quantified the percentage of the airway wall area relative to the total airway size for the six main segmental bronchi.

### Transcriptomic analysis

#### RNA extraction and normalisation

Whole blood samples were collected in PAXgene Blood RNA tubes, and total RNA was isolated using the Qiagen PreAnalytiX PAXgene Blood miRNA Kit (Qiagen, Valencia, CA).^30^^,^^32^ Samples were deemed suitable for sequencing if concentrations exceeded 25 μg/μL and the RNA integrity number (RIN) was greater than 6. The TruSeq Stranded Total RNA with Ribo-Zero Globin kit (Illumina Inc., San Diego, CA) was used to perform globin reduction, ribosomal RNA depletion, and cDNA library preparation, generating 75 base pair reads with an average of 20 million reads per sample on an Illumina HiSeq 2500 platform. TruSeq adapter sequences were removed using Skewer.^33^ Quality control was conducted with **FASTQC** (https://www.bioinformatics.babraham.ac.uk/projects/fastqc/) and **RNA-SeQC**.^34^ Reads were aligned to the human GRCh38 reference genome using STAR 2.5.^35^ Gene counts were generated using **RSubreads**^36^ with the **Ensembl** version 81 annotation.^37^

Genes with low expression were filtered out using the **filterByExpr** function in the **edgeR** package,^38^ retaining genes that had at least 20 counts in 50% or more of the samples. This step reduced noise and improved the reliability of the analysis by focusing on robustly expressed genes. Gene expression was normalised using the Trimmed Mean of M-values (TMM) method via the **calcNormFactors** function in **edgeR**, adjusting for differences in library sizes to ensure that expression levels were comparable across all samples.

#### Differential expression analysis

Differential expression analysis was performed using the **limma** (v3.58.1) and **edgeR** (v4.0.2) packages, with gene annotations retrieved using the **biomaRt** (v2.58.0) package in R.^38^ A design matrix was constructed using disease status while adjusting for age, sex, smoking status, and library construction batch. Batch effects were modelled as a covariate in the design matrix to isolate true biological differences from technical variations. Specific contrasts were defined using **makeContrasts** to compare disease groups against the control group and each other (e.g. COPD vs. Control, ACO vs. Control, Asthma vs. Control, ACO vs. COPD, and ACO vs. Asthma) and applied with **contrasts.fit**. We applied the **voom** transformation to the normalised data, converting counts to log-counts per million (logCPM) with precision weights. A linear model was fitted with **lmFit**, followed by empirical Bayes moderation (**eBayes**) to enhance statistical power and reliability. Significance was assigned at a p-value of 0.01, corrected for multiple testing using the Benjamini-Hochberg (BH) method to control the false discovery rate (FDR). For visualisation, the normalised counts were log-transformed using the **cpm** function, and batch effects were removed using the **removeBatchEffect** function to ensure accurate representation of biological variation in visual outputs, such as box plots.

### Enrichment analysis

#### GO enrichment

We utilised the **enrichGO** function from the **clusterProfiler** package (v4.10.0) with a significance threshold of 0.05, utilising **org.Hs.eg.db** (v3.18.0) for gene annotations and the Gene Ontology (GO) database for functional categorisation. The background gene set was defined as the refined gene set after excluding lowly expressed genes. Significant GO terms were hierarchically clustered based on functional similarities, assessed using **pairwise_termsim** function, and visualised in a tree diagram to highlight their relationships. In analysing upregulated differentially expressed genes, we limited enrichment analysis to gene sets containing 10–100 genes. The **circle_dat** function from the **GOPlot** library (v1.0.2) was employed to visualise genes to GO term connections. To improve visualisation clarity, we utilised the **reduce_overlap** function to reduce redundancy in gene-term links, with an overlap threshold set at 0.9.

#### KEGG enrichment and visualisation

The **enrichKEGG** function from **clusterProfiler** package was applied to ascertain statistically significant pathways (*P* ≤ 0.05). Gene identifiers were converted to a human-readable format using the **setReadable** function from the **org.Hs.eg.db** package. Enrichment scores were quantified by calculating the observed-to-expected ratio of differentially expressed genes within each pathway, normalised by the total gene count in each pathway. Pathways were then ranked based on adjusted p-values, gene count, and enrichment scores to identify statistically significant and biologically relevant enrichments. Pathway visualisation was performed using the **pathview** function (v1.42.0), which mapped gene expression data onto KEGG pathway diagrams, with genes colour-coded based on their expression levels.

### Statistics

For all analyses, listwise deletion was applied to address missing values, maintaining robust statistical power with over 2100 participants per analysis and enabling complete-case analysis using observed data. Multivariate linear regression analysis was conducted using cross-sectional data from the Phase 2 visit, ensuring all measurements were contemporaneous (i.e., no repeated measurements) and relevant to the current health status of the participants. The primary objective of the regression models was to quantify the independent associations between disease status (the exposure) and clinical outcomes, with adjustments made for potential confounders.

Given their established influence on respiratory outcomes, associations between PFT measures (excluding percent predicted features) and disease status were adjusted for age, sex, race, and height.^39^ Similarly, associations between CT features and disease status were adjusted for age, Body Mass Index (BMI), sex, race, smoking status, and scanner ID, considering their impact on respiratory and imaging outcomes.^31^ The estimated coefficients from fitted regression models reflect the direct association between disease status and clinical features, providing an accurate understanding of the effects of these diseases.

Disease status was included as a categorical variable with four levels: Control, Asthma, COPD, and ACO, with the Control group serving as the reference category. These categories were represented as separate indicator variables in the regression models, allowing for the estimation of distinct coefficients for each disease category while ensuring they were treated as exposures rather than mediators. Ordinary least squares (OLS) regression models were fitted using the **ols** function from the **statsmodels.formula.api** module of the Python **statsmodels** (v0.13.2) library.

To ensure the validity of the linear regression models, comprehensive diagnostic checks were conducted for key assumptions: normality, linearity, homoscedasticity, independence, and multicollinearity. Normality was assessed using the Kolmogorov–Smirnov (KS) test and by inspecting residual histograms and Q–Q plots. Given the large sample size, minor deviations from normality were unlikely to significantly impact results, as the Central Limit Theorem ensures residuals approximate normality in large datasets. Linearity was evaluated by plotting residuals against fitted values. Systematic patterns were observed, indicating a violation of the linearity assumption. Homoscedasticity was evaluated using the Breusch–Pagan test, which indicated significant heteroscedasticity. The independence of residuals was confirmed through the Durbin–Watson test, with values ranging between 1.5 and 2.5, indicating the absence of significant autocorrelation. Multicollinearity was assessed using the Variance Inflation Factor (VIF) for each predictor, with all VIF values remaining below the threshold of 10.

To address assumption violations, we applied Yeo-Johnson transformations to PFT features for variance stabilisation and quantile transformations to CT features to correct skewness and ensure normality with constant variance. Additionally, we performed 1000 bootstrap iterations to estimate regression coefficients, standard errors, confidence intervals, and residuals, and utilised Heteroskedasticity-Consistent (HC3) robust standard errors to correct for heteroscedasticity, improving the robustness of the model estimates. The standard errors of the regression coefficients were computed as the standard deviation of the bootstrap estimates, and 95% confidence intervals were derived using the 2.5th and 97.5th percentiles of the bootstrap estimates. P-values displayed in the forest plots were derived from bootstrapped regression models and adjusted using the BH correction. Overall differences among disease groups were identified using one-way ANOVA (P < 0.05), followed by Tukey's post-hoc test to determine specific pairs of disease categories with statistically significant differences. For each outcome variable, the predicted values from the regression model (fitted values) were inverse transformed to the original scale for clinical interpretability.

For symptom, exacerbation, PFTs (% predicted), and CBC variables, group differences were assessed using the Mann–Whitney U-test for continuous variables and the Chi-squared test for categorical variables, with Bonferroni correction applied to account for multiple comparisons. Using the Python **statannot** library (v0.2.3), we employed the **add_stat_annotation** function to annotate plots with statistical significance labels denoted as: ∗∗∗∗ for P < 0.0001, ∗∗∗ for P < 0.001, ∗∗ for P < 0.01, ∗ for P < 0.05, and ‘ns’ for non-significant.

### Role of funders

The content is solely the responsibility of the authors and does not represent the official views of the NHLBI or the NIH. The funding sources were not involved in the study design, data collection, data analyses, interpretation, or writing of this report.

## Results

### Clinical characteristics of COPDGene population

**Fig. 1:**
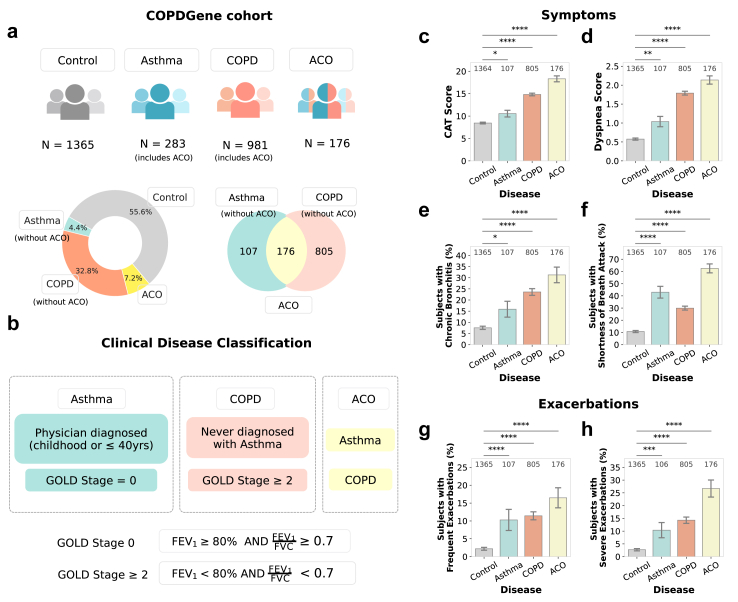
**Comprehensive characterisation of symptomatology and exacerbation patterns in the COPDGene Cohort. (a)** Distribution of total participants across control (N = 1365), asthma (N = 283, including those with overlapping COPD), COPD (N = 981, including those with overlapping asthma), and ACO (N = 176) clinical phenotypes (top). The left donut chart displays the proportion of participants classified within distinct clinical phenotypes, while the Venn diagram on the right depicts the overlap between asthma and COPD diagnoses, delineating the ACO group. **(b)** Clinical disease classification criteria based on GOLD stages and asthma diagnosis history, used to differentiate the phenotypes. **(c**–**h)** Comparative analysis of symptom severity and exacerbation patterns across clinical phenotypes. Bar plots displaying symptom severity as measured by **(c)** CAT scores and **(d)** mMRC Dyspnea scores across different participant groups. Bar plots showing the proportion of participants experiencing **(e)** chronic bronchitis, **(f)** shortness of breath attacks, **(g)** frequent exacerbations (requiring steroids and/or antibiotics per year), and **(h)** severe exacerbations (requiring emergency room visits or hospitalisations). Sample sizes for each group are indicated above the bars, and error bars represent 95% confidence intervals. Statistical analyses were conducted using the Mann–Whitney U-test for **(c)and(d)** and Chi-squared test for **(e–h)**, with Bonferroni correction applied for multiple comparisons. Statistical significance is annotated as: ns – not significant, ∗ P≤0.05, ∗∗ $P\leq 10^{-2}$, ∗∗∗ $P\leq 10^{-3}$, ∗∗∗∗ $P\leq 10^{-4}$.

The COPDGene study, one of the largest multicentre observational studies, consists of 10,192 participants, including smokers with and without COPD.^29^ As asthma is often co-diagnosed with COPD, individuals with asthma are included in COPDGene for an exhaustive representation of COPD. During the second phase (5-year follow-up), data were collected from 3925 clinically stable current and former smokers, at least 30 days post-exacerbation.^40^^,^^41^ Our analysis focused on a subset of 2453 subjects: 1365 smokers without a prior diagnosis of asthma and exhibiting normal spirometry constituted the control group; 238 individuals were classified with asthma (including subjects with concurrent COPD), 981 with COPD (including subjects with concurrent asthma); and 176 with both conditions—asthma and COPD (Fig. 1a). Participants with dual classifications were excluded from asthma and COPD groups, leading to final stratification into exclusive asthma (4.4%, N = 107), COPD-only (32.8%, N = 805), ACO (7.2%, N = 176), and smoking controls (55.6%, N = 1365) for all subsequent analyses.

The ‘smoking controls’ group comprises current and former smokers with a substantial smoking history (≥10 pack-years), without spirometric evidence of airflow obstruction, as defined by GOLD stage 0 (FEV_1_/FVC ratio ≥ 0.7 and FEV_1_ ≥ 80% predicted). This group was included as a baseline comparison to isolate disease-specific clinical and molecular effects from the broader effects of smoking. Importantly, the high prevalence of smoking controls underscores our focus on understanding disease-specific features within the context of smoking-related lung damage, without implying that smoking does not cause long-term harm.

Clinical phenotypes were determined based on physician diagnoses and spirometry,^41^ according to GOLD guidelines (Fig. 1b). Asthma was classified as a self-reported physician diagnosis before the age of 40 years, accompanied by normal spirometry. COPD was defined by an FEV_1_ below 80% and FEV_1_/FVC ratio below 0.7, corresponding to GOLD stages 2–4. ACO was defined as concurrent COPD with self-reported, physician-diagnosed asthma before the age of 40 years.^42^^,^^43^ Detailed demographic and clinical characteristics are summarised in Table 1.

**Table 1:** Characteristics of the COPDGene Study population.

Characteristic	Control	Asthma	COPD	ACO
Age (years)	63.49	61.71	68.68	64.34
BMI	29.05	29.26	27.81	29.26
**Sex (%)**				
Male	672 (49.23%)	36 (33.64%)	496 (61.61%)	76 (43.18%)
Female	693 (50.77%)	71 (66.36%)	309 (38.39%)	100 (56.82%)
**Race (%)**				
White	941 (68.94%)	65 (60.75%)	648 (80.5%)	116 (65.91%)
African American	424 (31.06%)	42 (39.25%)	157 (19.5%)	60 (34.09%)
**Smoking status (%)**				
Former	849 (62.2%)	59 (55.14%)	533 (66.21%)	106 (60.23%)
Current	516 (37.8%)	48 (44.86%)	272 (33.79%)	70 (39.77%)
**Gold stage (%)**				
Stage: 0.0	1365 (100.0%)	107 (100.0%)	0 (0.0%)	0 (0.0%)
Stage: 2.0	–	–	481 (59.75%)	111 (63.07%)
Stage: 3.0	–	–	233 (28.94%)	40 (22.73%)
Stage: 4.0	–	–	91 (11.3%)	25 (14.2%)

The demographic and clinical characteristics of the COPDGene Study participants, stratified into Control, Asthma, COPD, and ACO clinical phenotypes.

### ACO exhibits greater symptom severity and exacerbation rates among respiratory phenotypes

Comparative symptomatology (Methods, Supplementary Table S1) revealed the compounded burden of comorbid asthma and COPD within the ACO group. Symptom severity, as assessed through the CAT and mMRC Dyspnea Score, demonstrated a progressive increase from the control group through to the asthma, COPD, and ACO groups. The ACO group reported the highest CAT scores with a mean (M) score of 18.32 (P < 0.0001, MWU), followed by COPD (M = 14.83, P < 0.0001, MWU), asthma (M = 10.55, P < 0.05, MWU), and control (M = 8.45) subjects (Fig. 1c). The mMRC Dyspnea Score mirrored this trend, showcasing escalating breathlessness severity from control to the ACO group (Control: M = 0.58; Asthma: M = 1.04, P < 0.01; COPD: M = 1.79, P < 0.0001; ACO: M = 2.14, P < 0.0001; MWU) (Fig. 1d).

Focusing on chronic conditions associated with COPD, chronic bronchitis was most prevalent in the ACO at 31.25% (P < 0.0001, $\chi^{2}$), followed by the COPD at 23.60% (P < 0.0001, $\chi^{2}$), asthma at 15.89% (P < 0.05, $\chi^{2}$), and control group at 7.55% (Fig. 1e). However, emphysema prevalence was highest in COPD (15.80%, P < 0.0001, $\chi^{2}$), comparable to ACO (15.34%, P < 0.0001, $\chi^{2}$), and low in asthma (1.89%) and controls (4.69%) (Supplementary Figure S1a).

Dynamic episodic symptoms, particularly associated with asthma, such as shortness of breath attacks and wheezing, were most pronounced in the ACO group (Fig. 1f, Supplementary Figure S1b). The incidence of shortness of breath attacks was highest in ACO (62.50%, P < 0.0001, $\chi^{2}$), with declining prevalence in asthma (42.99%, P < 0.0001, $\chi^{2}$), COPD (29.94%, P < 0.0001, $\chi^{2}$), and the control group (10.77%), showcasing a severity gradient. Similarly, wheezing prevalence was greatest in ACO (78.41%, P < 0.0001, $\chi^{2}$), decreasing through asthma (60.75%, P < 0.0001, $\chi^{2}$), COPD (56.27%, P < 0.0001, $\chi^{2}$), to the control group (28.06%), signifying its presence across the spectrum of smoking-related respiratory conditions.

The prevalence of frequent exacerbations was lowest in the control group (2.20%, P < 0.0001, $\chi^{2}$), escalating through the asthma (10.28%, P < 0.0001, $\chi^{2}$), COPD (11.43%, P < 0.0001, $\chi^{2}$), and reaching the highest in ACO (16.48%, P < 0.0001, $\chi^{2}$), underscoring the progressive complexity of respiratory disease presentations (Fig. 1g). Severe exacerbations were most frequent in ACO at 26.70% (P < 0.0001, $\chi^{2}$), followed by COPD at 14.29% (P < 0.0001, $\chi^{2}$), asthma at 10.38% (P < 0.001, $\chi^{2}$), and the control group at 2.71%, highlighting a severity gradient peaking in ACO (Fig. 1h).

The LLN-based sensitivity analysis confirmed the robustness of the GOLD classification, as symptom and exacerbation patterns remained consistent despite the reclassification of 123 subjects (1 control to COPD, 106 unclassified from COPD, and 17 unclassified from ACO). Demographic details and analysis statistics are provided in Supplementary Table S1.

These patterns delineate a continuum of respiratory symptomatology and exacerbation frequency. Asthma is characterised by episodic and reversible symptoms, such as wheezing and shortness of breath, generally associated with lower exacerbation rates. In contrast, COPD exhibits more severe and persistent symptoms, including higher rates of chronic bronchitis, emphysema, and frequent, severe exacerbations, reflecting the chronic and progressive nature of the disease associated with advanced lung damage. ACO emerges as the most severe and heterogeneous phenotype, combining the airway hyperreactivity and episodic symptoms of asthma with the irreversible airflow limitation and frequent exacerbations seen in COPD. As a result, ACO experiences the highest rates of severe symptoms and exacerbations, bearing the most substantial clinical burden and underscoring the significance of this overlap syndrome in the management of chronic respiratory diseases.

### ACO and COPD subjects exhibit worse pulmonary function and gas exchange than asthma

**Fig. 2:**
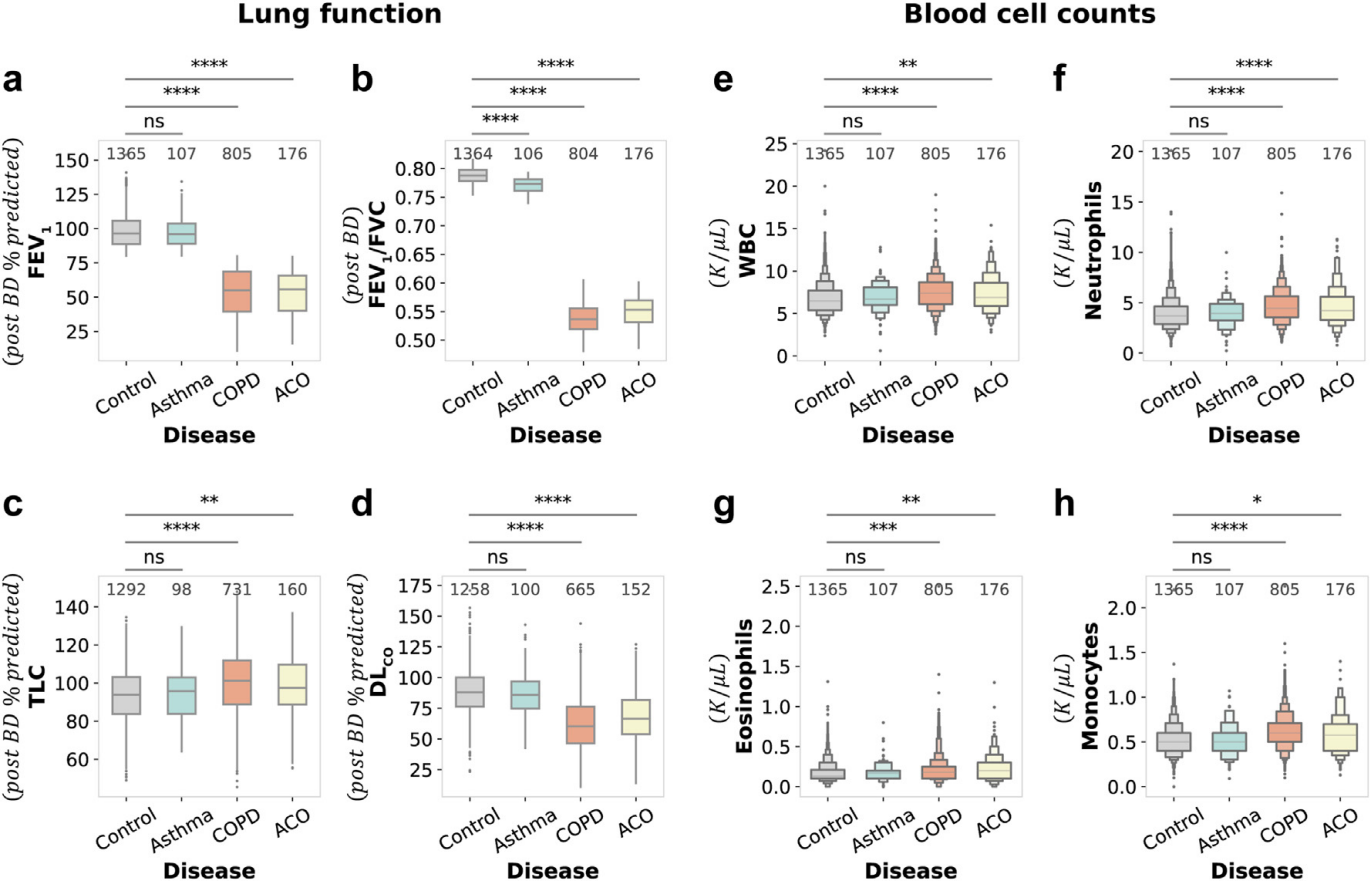
**Comparative analysis of pulmonary function tests and complete blood counts in COPDGene Cohort. a–d** Boxplot representing post-bronchodilator (post-BD) lung function parameters: **(a)** Percentage of predicted FEV_1_, **(b)** FEV_1_/FVC ratio (adjusted for confounders), **(c)** Percentage of predicted TLC, and **(d)** Percentage of predicted DL_CO_. Panels **e–h** illustrate blood cell counts: **(e)** WBC, **(f)** Neutrophils, **(g)** Eosinophils, and **(h)** Monocytes. Each boxplot denotes the interquartile range (IQR), with the median value represented by the horizontal line within the box. Whiskers extend to the furthest data points that are not considered outliers, and individual outliers are represented as dots. Mann–Whitney U test was used for panels **(a)**, **(c)**, **(d)**, and **(e**–**h)**, with Bonferroni correction applied for multiple comparisons. For the FEV_1_/FVC ratio, adjusted for confounders, in panel **(b)**, pairwise comparisons were performed using ANOVA followed by Tukey's HSD test. Sample sizes for each group are indicated above the boxplots, with significance levels annotated as follows: ns for non-significant, ∗ P≤0.05, ∗∗ $P\leq 10^{-2}$, ∗∗∗ $P\leq 10^{-3}$, ∗∗∗∗ $P\leq 10^{-4}$.

We analysed key pulmonary function tests to provide a multidimensional assessment of pulmonary health, covering airflow limitation, lung volume, gas exchange, and oxygenation, characteristic of various pulmonary disorders (Methods, Supplementary Table S1). Diagnostic plots evaluating the assumptions of linear models for FEV_1_/FVC and resting SaO_2_ are presented in Supplementary Figure S2. Notably, there was a marked decline in % predicted FEV_1_ from the control (M = 98.09%) to the asthma group (M = 96.93%), with a more substantial decrement evident in COPD (M = 53.37%, P < 0.0001, MWU) and ACO (M = 52.50%, P < 0.0001, MWU) groups, indicating severe airflow restriction (Fig. 2a). Similarly, the FEV_1_/FVC ratio was significantly higher in control subjects (0.79) than in asthma (0.77, P < 0.0001, HSD), COPD (0.54, P < 0.0001, HSD), and ACO (0.55, P < 0.0001, HSD) groups, underscoring obstructive lung pathology (Fig. 2b, Supplementary Figure S3a).

The % predicted TLC showed no significant deviation between control (M = 93.30%) and asthma (M = 94.45%) groups yet was notably higher in ACO (M = 98.34%, P < 0.01, MWU) and COPD (M = 100.27%, P < 0.0001, MWU) groups, suggesting lung hyperinflation and restrictive lung pathology (Fig. 2c). DL_CO_ percentages were markedly reduced in COPD (M = 61.71%) and ACO (M = 68.15%) compared to the control (M = 88.49%) and asthma (M = 87.29%) groups, reflecting significant gas exchange impairment (P < 0.0001, MWU) (Fig. 2d). Resting SaO_2_ levels were optimal in controls (97.18%) but slightly reduced in asthma (97.16%) and significantly lower in COPD (M = 95.78%, P < 0.0001, HSD) and ACO (M = 95.92%, P < 0.0001, HSD), highlighting potential hypoxaemia and more severe gas exchange deficits (Supplementary Figure S3b and c).

Asthma generally exhibits mild functional impairment, with near-normal FEV_1_ (% predicted) and a slightly reduced FEV_1_/FVC ratio, reflecting mild airflow limitation that is largely reversible. The preserved DL_CO_ suggests minimal alveolar–capillary interface damage, and normal TLC indicates that hyperinflation is not a significant feature, aligning with the absence of significant emphysema. Despite a smoking history, asthma maintains better lung function with less functional damage compared to ACO and COPD. ACO shows FEV_1_ and FEV_1_/FVC ratio nearly identical to COPD, indicating similarly severe and largely irreversible airflow limitation. However, ACO retains slightly better DL_CO_ and SaO_2_ than COPD, suggesting partial preservation of alveolar function, likely influenced by the asthma component. Despite this, ACO still exhibits significant lung hyperinflation (TLC) and reduced gas exchange capacity compared to asthma. This combination of both obstructive and partially reversible traits in ACO, alongside the severity of impairment seen in COPD, reveals the heterogeneity of ACO. This comparison highlights the progression from mild impairment in asthma to moderate in ACO and severe dysfunction in COPD, underscoring the distinct yet overlapping pathophysiological features of these diseases.

LLN criteria displayed similar trends, with ACO and COPD exhibiting worse pulmonary function and gas exchange compared to asthma, though ACO retained slightly preserved lung function and gas exchange than COPD (Supplementary Table S1).

### Elevated leukocyte concentrations reflect enhanced systemic inflammation in ACO and COPD

The CBC data (N = 2453) underscored variance in systemic inflammation among distinct respiratory conditions (Methods, Supplementary Table S1). Total white blood cell (WBC) counts were notably higher in COPD (7.49 K/μL, P < 0.0001, MWU) and ACO (7.35 K/μL, P < 0.01, MWU) groups compared to control (6.73 K/μL) group, with asthma showing similar levels to the control (6.90 K/μL) group (Fig. 2e). Neutrophil concentrations, suggestive of neutrophilic inflammatory response, were significantly elevated in COPD (4.68 K/μL, P < 0.0001, MWU) and ACO (4.57 K/μL, P < 0.0001, MWU) over controls (3.92 K/μL), while asthma showed neutrophil counts similar to controls (4.02 K/μL) (Fig. 2f).

Eosinophil levels escalated from control (0.17 K/μL) through asthma (0.18 K/μL) to reach their zenith in COPD (0.20 K/μL, P < 0.001, MWU) and ACO (0.22 K/μL, P < 0.01, MWU), with inflammation particularly prominent in ACO (Fig. 2g). Elevated monocyte counts in COPD (0.61 K/μL, P < 0.0001, MWU) and ACO (0.59 K/μL, P < 0.05, MWU) against control (0.53 K/μL) group highlighted their role in sustained inflammation (Fig. 2h). Lymphocyte numbers, conversely, were lower in COPD (1.94 K/μL, P < 0.001, MWU) and ACO (1.93 K/μL) compared to controls (2.07 K/μL) and asthma (2.13 K/μL), hinting at an immunological shift in chronic respiratory diseases (Supplementary Figure S4a).

Leukocyte correlations further delineated unique inflammatory signatures across diseases, with neutrophils showing a strong link to total WBC counts, particularly in ACO (Supplementary Figure S4b–e). A higher eosinophil-WBC correlation in asthma emphasised the role of eosinophils in allergic inflammation, contrasting with their lower correlation in COPD and control groups. Monocytes demonstrated a steady correlation with WBCs in all conditions, indicating their ubiquitous influence, whereas lymphocytes exhibited the lowest correlations in COPD and ACO, pointing to divergent regulation across conditions. Furthermore, a low eosinophil-neutrophil correlation across disease groups suggests independent regulation of these cell types. The analysis underscores the systemic inflammation characterising COPD and ACO, with distinct leukocyte profiles and correlation patterns, shedding light on the intricate immune dynamics within respiratory disorders.

Overall, COPD is marked by elevated WBC, neutrophil, and monocyte counts, indicating a strong systemic inflammation consistent with chronic and progressive nature of the disease. Asthma displayed the mildest inflammation, with leukocyte counts similar to controls, reflecting its episodic symptoms and preserved lung function, where airflow limitation is mild and reversible. ACO presents a unique inflammatory pattern, with elevated neutrophils and highest eosinophils, emphasising distinct immune mechanisms across phenotypes. LLN criteria confirm systemic inflammation in COPD and ACO, and its relative absence in asthma (Supplementary Table S1).

### COPD and ACO showcase complex interplay of innate and adaptive immune mechanisms

Building on PFT findings, we performed DE analysis using bulk RNA-seq data (N = 2453) to delineate cellular mechanisms underpinning disease aetiology in clinical phenotypes as compared with smoking controls. Adjusting for age, sex, smoking status, and library construction batch, the analysis revealed no differentially expressed genes (DEGs) in asthma, implying equivalent gene expression to controls. Conversely, significant transcriptomic changes were observed in COPD (1271 upregulated, 1322 downregulated genes) and ACO (604 upregulated, 714 downregulated genes) at an FDR of 0.01, highlighting distinct molecular pathologies (Methods, Supplementary Table S2).

**Fig. 3:**
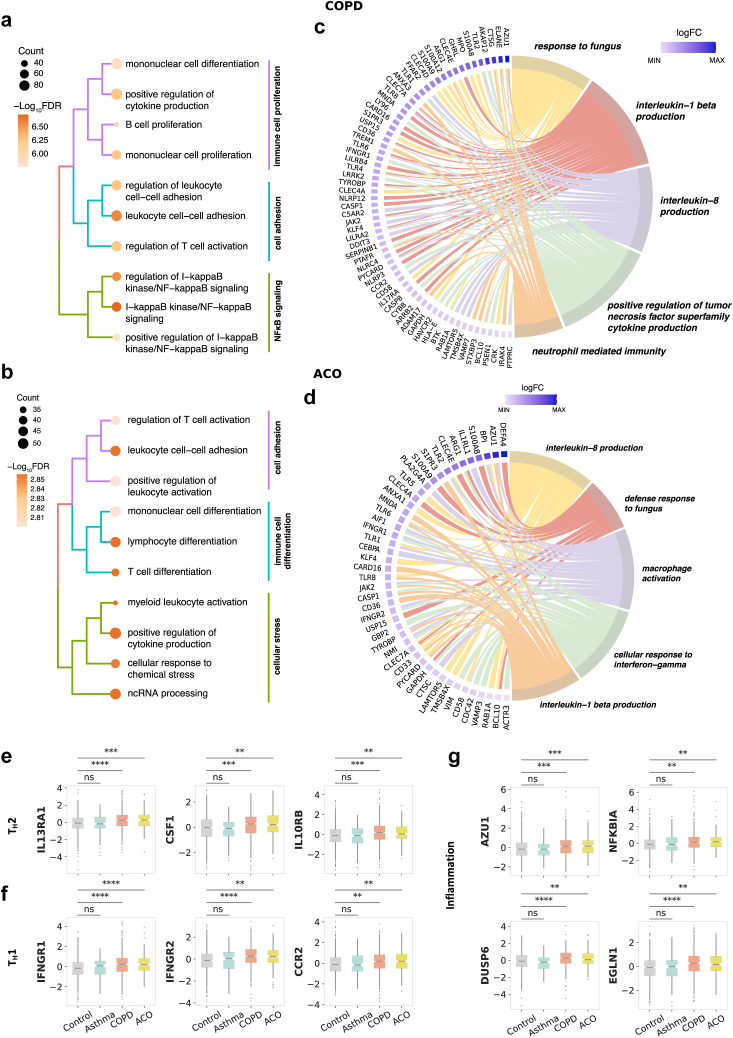
**Functional enrichment and Gene Expression Profiling in COPD and ACO. (a)** Tree plot showing Gene Ontology (GO) enrichment analysis of biological process annotations in COPD, displaying the top 10 most statistically significant GO terms, clustered into three primary categories. The size of dots represents the gene count of differentially expressed genes (DEGs) associated with each GO term, while the colour indicates -log_10_ FDR value. **(b)** Tree plot of GO term enrichment analysis for ACO, displaying the top 10 significant categories of GO terms, organised into three distinct clusters. **(c)** Circos plot showing the association between upregulated DEGs in COPD and top 5 GO biological processes. The colour gradient indicates the log_2_ fold change for each gene. **(d)** Circos plot illustrating the mapping of upregulated DEGs in ACO to the top 5 GO categories. In panels **(a**–**d)**, top categories in COPD and ACO are coloured by the rank based on statistical significance rather than nomenclature, as they may correspond to different gene sets. **(e)** Box plots showing the expression levels of upregulated genes involved in cytokine production in COPD and ACO. **(f)** Box plots depicting the expression levels of upregulated genes involved in T-cell activation in COPD and ACO. **(g)** Box plots highlighting the differential expression of upregulated inflammatory and immune response genes in COPD and ACO. Statistical significance is denoted by asterisks (ns = not significant, ∗ P≤0.05, ∗∗ $P\leq 10^{-2}$, ∗∗∗ $P\leq 10^{-3}$, ∗∗∗∗ $P\leq 10^{-4}$). This figure illustrates the distinct molecular signatures of immune response in COPD and ACO.

GO overrepresentation analysis for Biological Processes (BP) revealed a shared immunological landscape characterised by cytokine production and leukocyte adhesion in both COPD and ACO (Supplementary Table S3). In COPD, a robust upregulation of genes involved in immune cell proliferation, notably within mononuclear and B cell populations (Fig. 3a). This finding underscores a potential hyperactivity within the adaptive immune system, which may drive the persistent inflammatory state characteristic of COPD. Furthermore, we identified enrichment of genes regulating leukocyte cell–cell adhesion and T cell activation, suggesting that aberrant immune cell interactions are a hallmark of COPD pathophysiology. Significant enrichment in the Nuclear Factor Kappa B (NF-κB) signalling pathway was also evident, reaffirming its pivotal role in mediating inflammatory responses in COPD.

The GO BP analysis in ACO group was dominated by immune processes, including cell adhesion, immune cell differentiation, and cellular stress responses (Fig. 3b), indicating an intricate interaction of immune responses, with a distinct emphasis on adaptive immunity and myeloid cell involvement. A notable emphasis on the activation and differentiation of T cells and myeloid leukocytes, along with cytokine production, suggested a diverse immune response, potentially reflecting the mixed pathology of ACO.

Upon identifying broader immune categories of biological processes, we performed a detailed investigation of elevated immune responses emanating from upregulated genes (Fig. 3c, Supplementary Table S3). In COPD, we identified immune pathways associated with response to fungus ($P=2.30\times 10^{-5}$), interleukin-1 beta production ($P=9.93\times 10^{-5}$), interleukin-8 production ($P=4.25\times 10^{-4}$), positive regulation of tumor necrosis superfamily cytokine production ($P=4.25\times 10^{-4}$), neutrophil mediated immunity ($P=5.88\times 10^{-3}$) (all p-values from hypergeometric test, BH corrected). These processes were associated with upregulation of innate immunity and pattern recognition genes, *TYROBP*, *LILRB4*, *LILRA2*, *LY96*, toll-like receptor (*TLR*) genes *TLR1*, *TLR4*, *TLR6*, *TLR8*, c-type lectin genes *CLEC7A*, *CLEC4E*, *CLEC4A*, *CLEC4D*, CD36, B cell receptor signalling genes *BCL10*, *BTK*, *CRK*, *JAK2*, *CARD16*, *MNDA*, *IRAK4*, *HAVCR2*, *AKAP12*, neutrophil production genes including *AZU1*, *ELANE*, *CTSG*, *ARG1*, and *MPO*, inflammasome assembly genes *NLRP3*, *NLRC4*, and *PYCARD*, and tissue remodelling and repair genes *ADAM17*, *TREM1*, *ANXA3*.

In ACO, the BPs associated with upregulated genes included interleukin-8 production ($P=1.02\times 10^{-5}$), defence response to fungus ($P=8.11\times 10^{-4}$), macrophage activation ($P=8.11\times 10^{-4}$), cellular response to interferon-gamma ($P=8.11\times 10^{-4}$), interleukin-1 beta production ($P=4.86\times 10^{-3}$) (all p-values from hypergeometric test, BH corrected) (Fig. 3d, Supplementary Table S3). These BPs annotated to innate immunity and pattern recognition genes belonging to TLR family *TLR1*, *TLR2*, *TLR5*, *TLR6*, *TLR8*, and c-type lectin genes *CLEC7A*, *CLEC4E*, *CLEC4A*, cytokine production and regulation genes *KLF4*, *S1PR3*, *MNDA*, *IFNGR1*, *IFNGR2*, *IL1RL1*, neutrophil function and activation genes *BPI*, *DEFA4*, *AZU1*. The expression of prominent cytokine signalling genes *TGFBR1*, *CSF1*, *TNFSF10*, T cell signalling genes *IFNGR1*, *IFNGR2*, *CCR2*, inflammation markers like *AZU1* and *NFKBIA* and hypoxia-associated genes *DUSP6* and *EGLN1* across disease groups are provided in Fig. 3e–g. LLN-based classification revealed similar categories of pathways in COPD and ACO, reinforcing the shared molecular mechanisms in these respiratory conditions (Supplementary Table S2, S3).

Collectively, our study reveals that COPD and ACO involve complex and nuanced interactions between the innate and adaptive immune systems, reflecting the intricate immunological landscape underpinning these respiratory conditions.

### COPD and ACO share NF-κB-driven inflammatory pathways with ACO uniquely enriched in HIF-1A signalling and COPD in neutrophil extracellular trap formation and necroptosis

KEGG pathway enrichment (FDR = 0.05) highlighted a central role of NF-κB pathway mediated inflammation in COPD ($P=4.78\times 10^{-4}$) and ACO ($P=3.44\times 10^{-3}$) (both p-values from hypergeometric test, BH corrected), with ACO showing upregulation of NF-κB signalling genes *LY96*, *TRAF1*, *TRAF5*, *BTK*, *NFKBIA*, and *CHUK* (Supplementary Table S3, Supplementary Figure S5a). Elevated *LTBR* expression could amplify lymphocyte-driven inflammation in ACO, while varied expression of apoptosis regulators (*BCL2A1*, *BCL2*), stress response (*GADD45A*), and DNA damage (*ATM*, *PARP1*) genes suggests an intricate balance between cell survival and inflammation. Furthermore, changes in kinases (*PRKCQ*) and T-cell signalling (*ZAP70*, *PLCG1*, *CARD11*) hint at disrupted cell damage control and increased immune response.

In COPD, genes related to the NF-κB pathway and immune regulation, such as *BCL2A1*, *LY96*, *TNFSF13B*, *TLR4*, indicated an enhanced inflammatory response (Supplementary Figure S5b). The expression changes in *TRIM25*, *CFLAR*, *TNFRSF1A*, and the upregulation of *NFKBIA*, *BTK*, *CHUK*, *BCL10* suggest a pro-inflammatory network, while downregulation of *TRAF3*, *CSNK2A2*, and T-cell signalling molecules hints at disrupted immune signalling.

**Fig. 4:**
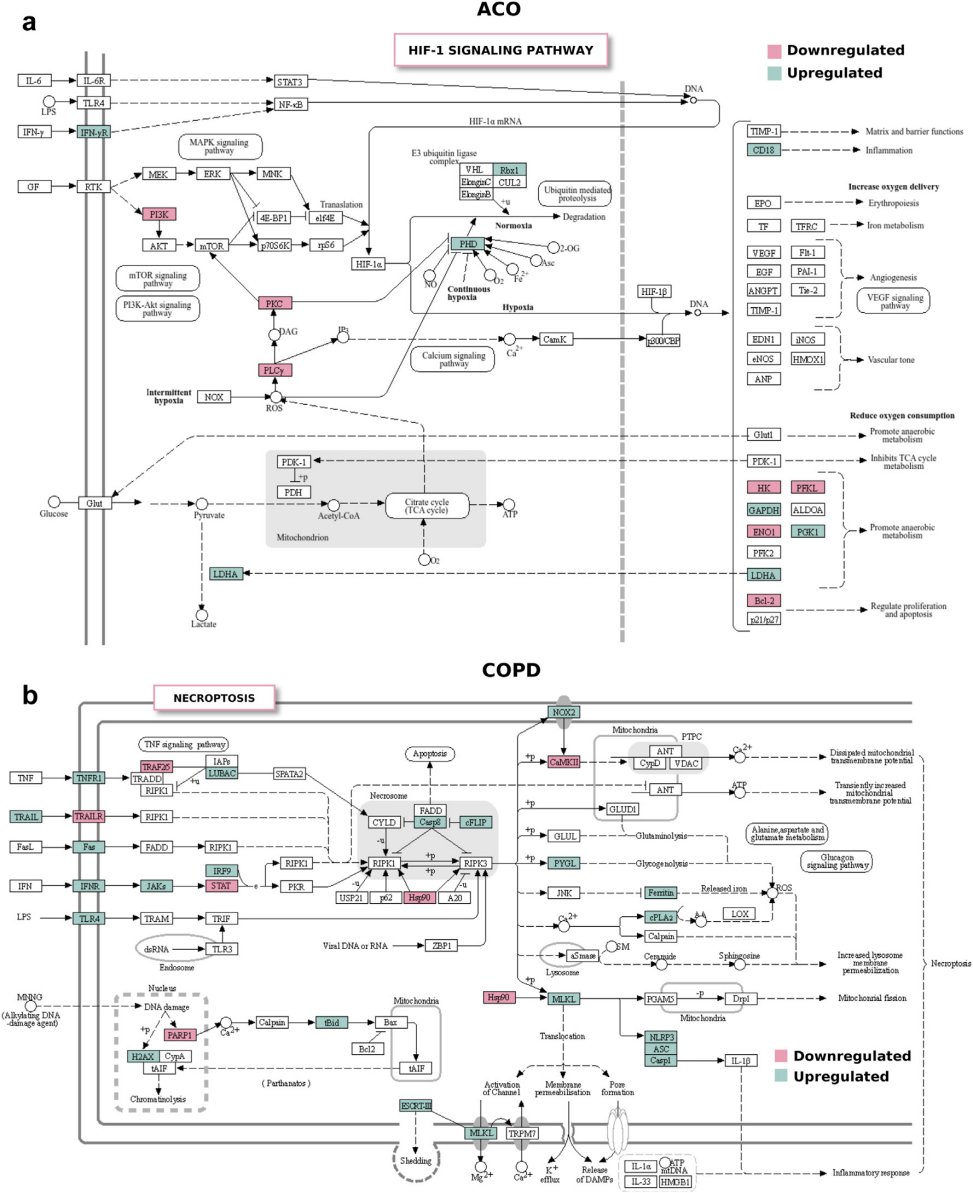
**Pathway activation in ACO and COPD identified through KEGGpathway enrichmentanalysis.** Pathway maps displaying **(a)** The HIF-1 signalling pathway enriched in ACO. **(b)** Necroptosis pathway enriched in COPD. In **(a** and **b)**, upregulated pathway components are marked in pink, while downregulated components are marked in green. These diagrams depict the molecular mechanisms underlying hypoxic adaptation in ACO and cell death regulation in COPD, providing insights into the distinct and overlapping biological processes that contribute to the pathophysiology of these conditions.

Interestingly, ACO exhibited unique enrichment in amino and nucleotide sugar metabolism pathways, along with the HIF-1 signalling pathway, suggesting significant metabolic reprogramming and adaptation to hypoxic conditions (Fig. 4a, Supplementary Table S3). Upregulation of HIF-1 signalling genes associated with glycolysis and immune responses under low oxygen conditions, including *IFNGR1*, *GAPDH*, *IFNGR2*, *EGLN1*, *PGK1*, *LTBR*, *RBX1*, *HK3*, and *LDHA*, indicates cellular adaptation to hypoxia. *LDHA* facilitates anaerobic glycolysis, converting pyruvate to lactate to maintain ATP production under hypoxia. *HK3*, which is crucial for glucose metabolism by phosphorylating glucose to glucose-6-phosphate—a key step in glycolysis and energy production—was upregulated in fructose and mannose metabolism, amino sugar and nucleotide sugar metabolism, and biosynthesis of nucleotide sugars. Additionally, upregulation of *UGP2*, *GFUS*, and *PGM2* in both amino sugar and nucleotide sugar metabolism and biosynthesis of nucleotide sugars pathways, supports increased nucleotide biosynthesis, aligning with the metabolic shift driven by HIF-1 signalling. This coordinated upregulation enhances cell survival and function in hypoxic environments by boosting glycolytic flux and nucleotide production.

Notably, COPD exhibited a unique enrichment of neutrophil extracellular trap (NET) formation ($P=1.98\times 10^{-6}$), and necroptosis ($P=6.70\times 10^{-6}$) pathways (both p-values from hypergeometric test, BH corrected), highlighting the distinct mechanistic underpinnings of its inflammatory cascade (Supplementary Figure S6). Key genes associated with NET structure, including *AZU1*, *ELANE*, *CTSG*, and *MPO*, were significantly upregulated, suggesting an aggressive defence mechanism that may lead to tissue damage. The enhanced expression of pathogen recognition genes *TLR2* and *TLR4* potentially amplifies NETosis. Furthermore, histone genes *H3C8*, *H2AC4*, and *H3C3*, along with NADPH oxidase complex components *NCF1*, *NCF2*, *NCF4*, and *CYBB*, underscore the involvement of reactive oxygen species and chromatin remodelling in NET formation. Activation of the inflammasome is indicated by the upregulation of *CASP1* and *CASP4*, while the downregulation of signalling genes *PIK3CA*, *PIK3R1*, *AKT3*, and *PLCG1* suggests a dysregulated inflammatory response in COPD, highlighting a complex interplay of neutrophil activation and immune signalling.

In COPD, the necroptosis pathway enrichment suggests a complex inflammatory response, involving dysregulated genes associated with this form of programmed cell death (Fig. 4b). Key upregulated genes initiating necroptosis include *TNFSF10*, *FAS*, and *TNFRSF1A*, *alongside MLKL*, indicating an active process disrupting cell membranes. Oxidative stress is highlighted by *CYBB*, part of the NADPH oxidase complex, while inflammasome components *NLRP3*, *ASC*, and *CASP1* link inflammation to necroptosis. Genes like *PGAM5* and *DRP1* suggest necroptosis enrichment, impacting mitochondrial integrity and potentially affecting airway structure and repair. Histone genes, including *H2AC4*, *H2AC14*, *H2AC12*, *H2AC13*, *H2AC16*, *H2AC21*, *H2AZ1*, and *H2AC6*, along with ESCRT-III complex components *CHMP5*, *CHMP1B*, *CHMP3*, *CHMP2B*, and *CHMP7*, underscore the role of chromatin remodelling and cell membrane repair in necroptosis. This active cell death process could worsen COPD pathophysiology by promoting airway remodelling and impairing tissue repair.

Under LLN criteria, ACO retained enrichment in amino and nucleotide sugar metabolism pathways, emphasising a metabolic shift without strong evidence for HIF-1 involvement. Similarly, COPD consistently showed enrichment in NETosis and necroptosis pathways, reinforcing the pivotal role of neutrophil-driven inflammation in COPD (Supplementary Table S4).

In summary, both COPD and ACO are driven by NF-κB-mediated inflammation. ACO is uniquely enriched in HIF-1 signalling and metabolic pathways related to hypoxic adaptation, while COPD shows enrichment in neutrophil extracellular trap formation and necroptosis, contributing to its distinct inflammatory profile and tissue damage.

### CT scan analysis reveals predominant parenchymal destruction in COPD, severe airway remodelling in ACO, and superior lung preservation in asthma

**Fig. 5:**
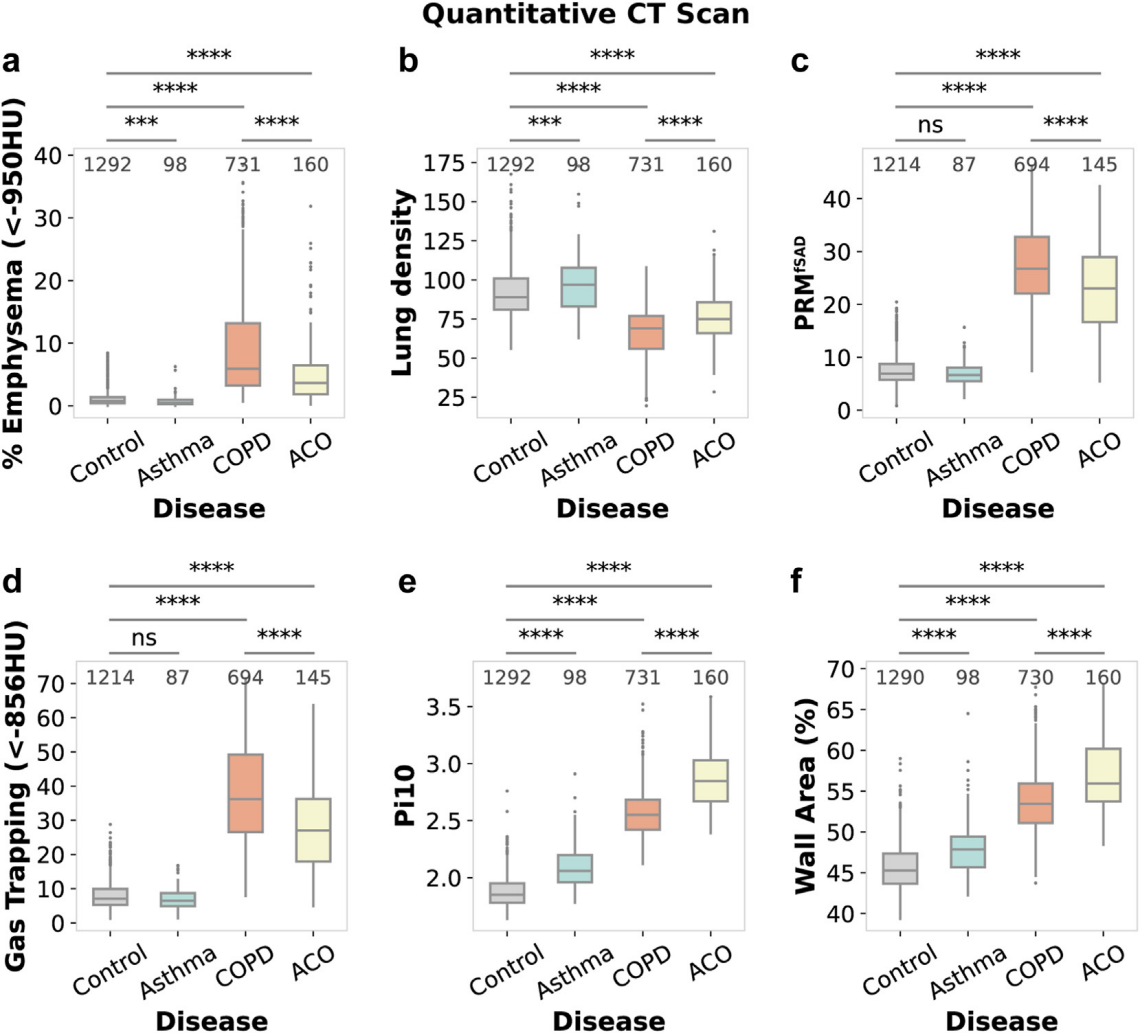
**Comparative analysis of quantitative CT scan-derived pulmonary metrics across different respiratory conditions.** Boxplots displaying **(a)** percentage of emphysema determined by low attenuation areas (<−950 HU), **(b)** lung density measured at the 15th percentile (Perc15), **(c)** Parametric Response Mapping for small airways disease (PRM^fSAD^), **(d)** gas trapping quantified by low attenuation at −856 HU, **(e)** Pi10 measurement, reflecting standardised airway wall thickness, **(f)** airway wall area percentage as an indicator of airway remodelling. Each boxplot represents the interquartile range, with median values marked by horizontal lines. Whiskers extend to the most extreme data points not considered outliers, and outliers are plotted as distinct points. The sample size for each disease phenotype is noted above the respective boxplots. Statistical significance was determined using ANOVA followed by Tukey's HSD test, with significant differences between disease groups annotated as follows: ns = not significant, ∗ P≤0.05, ∗∗ $P\leq 10^{-2}$, ∗∗∗ $P\leq 10^{-3}$, ∗∗∗∗ $P\leq 10^{-4}$.

CT scan evaluations within the COPDGene Study revealed pronounced structural alterations in COPD and ACO, with LLN-based classification capturing a similar overall pattern of structural damage as observed with GOLD classification (Methods, Supplementary Table S1). Diagnostic plots evaluating the assumptions of linear models used in the analysis are presented in Supplementary Figure S7. Emphysema quantification based on low attenuation areas (below −950 HU) highlighted extensive lung tissue destruction in COPD (8.90%, P < 0.0001, HSD) and ACO (5.66%, P < 0.0001, HSD) compared to the control group (1.09%) and lower involvement in asthma (0.80%, P < 0.001, HSD), with COPD displaying higher emphysema than ACO (P < 0.0001) (Fig. 5a, Supplementary Figure S8a). The 15th percentile lung density (Perc15) showed notable change, with an increase in lung density from the control group (92.11%) to asthma (98.72%, P < 0.001, HSD); and decrease in ACO (75.90%), and COPD (66.30%), indicating significant emphysematous tissue loss in COPD and ACO, although ACO showed less severe decline than COPD (P < 0.0001, HSD) (Fig. 5b, Supplementary Figure S8b).

Using Parametric Response Mapping (PRM) on CT scans, we identified a significant increase in non-functional small airway disease (fSAD) areas indicating air trapping in COPD (27.09%) and ACO (22.94%) compared to the control group (7.57%), with no significant difference in asthma (7.00%) (P < 0.0001, HSD). COPD showed more severe small airway disease than ACO (P < 0.0001, HSD), highlighting greater airway obstruction (Fig. 5c, Supplementary Figure S5c). CT imaging at −856 HU attenuation revealed significant post-expiratory air retention, with elevated gas trapping in COPD (37.34%) and ACO (28.86%) compared to controls (7.96%), with COPD showing a more pronounced effect than ACO (P < 0.0001, HSD), while asthma's mean gas trapping (7.25%) was slightly lower but not significantly different from controls (Fig. 5d, Supplementary Figure S5d). This suggests non-functional, trapped air regions within the lungs, consistent with the obstructive pathology of these conditions.

Airway wall thickness, assessed through Pi10 values, demonstrated increased remodelling from controls (1.87) through asthma (2.09 mm) to COPD (2.57 mm) and peaking in ACO (2.87 mm) (P < 0.0001, HSD) (Fig. 5e, Supplementary Figure S5e). Additionally, airway wall area percentage indicated progressive remodelling, most severe in ACO (56.93%), compared to COPD (53.73%) and a slight elevation in asthma (48.13%) over controls (45.65%) (P < 0.0001, HSD) (Fig. 5f, Supplementary Figure S5f). Both airway wall thickness and the wall area percentage were highest in ACO, surpassing COPD, pointing to more pronounced airway remodelling in ACO.

In COPD, the lungs show widespread destruction with significant emphysema, air trapping, and small airway disease, reflecting progressive parenchymal damage and loss of lung function. Increased small airway disease (27.09%) and gas trapping (37.34%) contribute to airflow limitation, but the extensive emphysema also plays a central role by reducing the lung's ability to exchange gases. ACO, on the other hand, presents a combination of moderate emphysema, severe airway remodelling, and small airway disease (22.94% non-functional areas, 28.86% gas trapping). This combination reflects both parenchymal destruction and significant airway-centred damage. In contrast, asthma displays minimal emphysema, well-preserved lung parenchyma, and moderate airway thickening, primarily resulting in airway hyperresponsiveness rather than extensive tissue destruction. While small airway disease is less prominent in asthma, the airway remodelling seen in ACO (with the highest Pi10 and wall area percentage) suggests more severe obstruction due to structural changes. These findings imply that in COPD and ACO, airflow limitation is driven by a multifactorial interplay of small airway disease, airway remodelling, and emphysema, with asthma remaining predominantly airway focused.

## Discussion

**Fig. 6:**
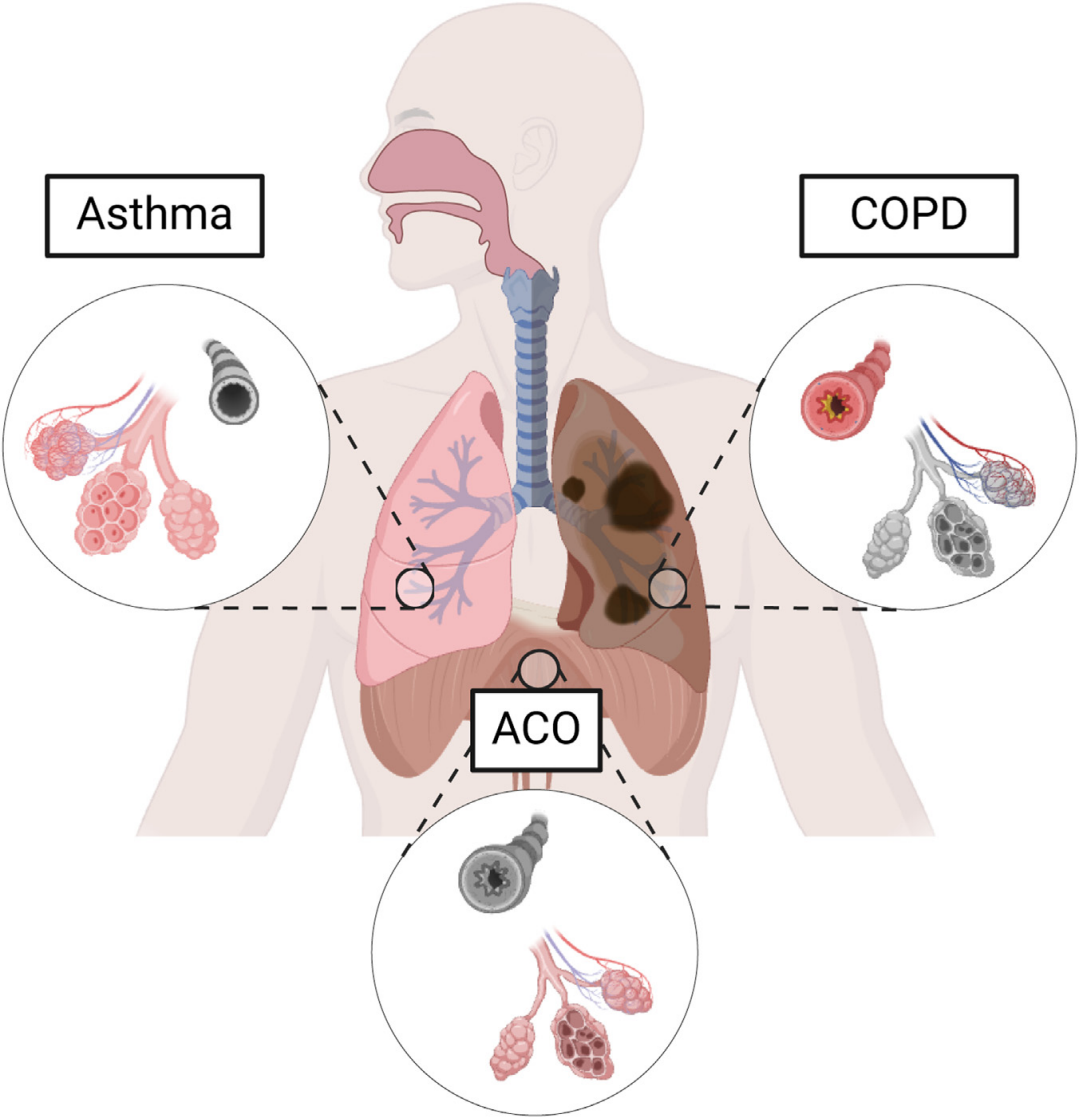
**Schematic overview of respiratory pathologies in asthma, COPD, and ACO,depictingthelung regionsaffectedin each condition.** The diagram illustrates the primary areas impacted by asthma (left), characterised by bronchial hyperresponsiveness and inflammation; COPD (right), marked by alveolar damage and airway obstruction; and ACO (centre), which exhibits features of both asthma and COPD, including airway inflammation and parenchymal destruction. This visual representation underscores the distinct and overlapping pathophysiological features of these chronic respiratory conditions. The schematic was generated using Biorender.com.

Given the overlapping aetiology and pathophysiology of asthma and COPD, there is a pressing need for clear diagnostic criteria for accurate ACO diagnosis.^15^^,^^44^^,^^45^ Existing definitions based on clinical features and biomarkers have sparked debate over whether ACO represents a distinct phenotype or merely a convergence of asthma and COPD, thereby complicating efforts to standardise diagnosis and treatment.^46^ We show that ACO represents a distinct clinical entity by combining episodic and reversible airway hyperresponsiveness of asthma with the progressive airflow obstruction and parenchymal destruction of COPD (Fig. 6). This combination leads to a more severe clinical presentation, marked by diverse symptoms, inflammation, molecular signatures, and morphological alterations that cannot be fully explained by either condition alone.

Our study focused on participants with significant smoking histories (≥10 pack-years), using smokers as controls to isolate disease-specific changes from the general effects of smoking. Additionally, by conducting measurements during the stable phase (≥30 days post-exacerbation), we minimised the confounding impact of acute inflammation on the disease pathology. Beyond GOLD classification criteria, we applied LLN criteria, which captured structural, clinical, and inflammatory patterns between respiratory phenotypes equally well (Supplementary Table S1), consistent with findings from a large study of over 24,000 individuals showing that a fixed FEV_1_/FVC < 0.7 predicts COPD outcomes as accurately as LLN.^47^

While the smoking controls, constituting a significant proportion of the cohort (55.6%), exhibit no detectable functional deficits according to spirometry, they remain at significant long-term risk due to smoking-induced inflammation and lung damage. Prior research has shown that even individuals with normal lung function can experience smoking-induced inflammation and structural damage,^48^ increasing the likelihood of future respiratory impairment. It is important to note that this group does not represent the broader U.S. population; their inclusion underscores the variability in smoking-related subclinical lung damage and the ongoing risks that persist despite preserved lung function.

Our findings demonstrate that asthma is primarily an airway-centred disease with episodic symptoms, reversible airflow limitation, preserved lung function, minimal systemic inflammation, and airway wall thickening, while COPD emerges as the most severe phenotype, marked by irreversible airflow limitation, neutrophil-driven inflammation, extensive parenchymal destruction, and prominent small airway disease contributing to airflow obstruction. Our study demonstrates that ACO, as a complex obstructive pulmonary disease, spans the asthma-COPD phenotypic spectrum by combining key elements of both diseases. ACO exhibits irreversible airflow limitation similar to COPD, with a significantly reduced FEV_1_/FVC ratio and impaired gas exchange, yet retains better lung function than COPD, as indicated by higher DL_CO_ values and less parenchymal destruction. Although ACO shares emphysema and small airway disease with COPD, it shows less parenchymal destruction, as evidenced by higher lung density and reduced gas trapping. However, ACO exceeds both asthma and COPD in terms of airway remodelling, displaying thicker airway walls and a greater wall area percentage, reflecting more extensive airway damage.

Elevated leukocyte counts, particularly a stronger eosinophil component and less dominant neutrophilic response compared to COPD, reflect a mixed inflammatory profile and systemic inflammation, contributing to the severe symptomatology. Previous studies have not only established a link between increased eosinophils and neutrophils and exacerbation risks in ACO,^49^^,^^50^ but also demonstrated therapeutic value of targeting eosinophils in ACO to reduce exacerbations,^51^ confirming the role of eosinophilic inflammation in ACO. This hybrid nature of ACO, encompassing features of both diseases but with distinct characteristics, underscores the need for a multifaceted therapeutic approach that targets airway hyperreactivity, structural changes, and inflammation.

The immune profile of ACO diverges from COPD by incorporating both innate and adaptive immune responses, with a notable emphasis on T cell activation and interferon-gamma pathways, unlike COPD, which is largely driven by innate mechanisms such as neutrophil activation and NF-κB signalling. Notably, asthma did not show significant transcriptomic deviations, likely because the molecular signatures of stable asthma closely resemble those of smoking controls without obstructive lung disease. Additionally, since asthma's inflammation is largely confined to the airways, it may not translate to detectable changes in peripheral blood during stable periods.

A previous COPDGene cohort study (N = 1954) identified unique alterations in the taste transduction pathway, particularly in T2R genes, involved in airway smooth muscle function, epithelial integrity, and immune response, with these changes more prominent in ACO than COPD.^41^ Using a larger subset of 2453 participants and at a stricter FDR (0.01 vs. 0.1) of identifying DE genes, our pathway enrichment analysis revealed that both diseases are driven by NF-κB mediated inflammation, aligning with the systemic inflammation observed in CBC and the structural alterations identified in CT scans. ACO exhibits distinct HIF-1 signalling and metabolic pathways, including amino and nucleotide sugar metabolism, reflecting shifts in energy production and immune responses. This differs from COPD's focus on necroptosis and NET formation, processes that drive tissue damage, exacerbate inflammation, and contribute to the more pronounced structural lung damage and functional decline observed in COPD compared to ACO.

HIF-1α signalling, by promoting glycolysis and limiting mitochondrial respiration, is known to help lung tissues adapt to chronic hypoxia but may also enhance fibrotic and remodelling processes,^52^^,^^53^ leading to the thickened airways and small airway disease characteristic of ACO. This protective but maladaptive response may also contribute to less severe emphysema compared to COPD, as tissue remodelling limits further parenchymal destruction. NETosis is established to be associated with disease severity in COPD patients,^54^, ^55^, ^56^ supported by the observed GO enrichment of neutrophil-mediated immunity (Fig. 3C). NET formation, characterised by the release of chromatin and antimicrobial proteins by neutrophils, is a defence mechanism gone awry in COPD, contributing to inflammation and tissue damage. The involvement of necroptosis, a programmed form of inflammatory cell death, introduces an additional layer of complexity to COPD's pathology, potentially exacerbating airway remodelling and impairing tissue repair. Similarly, the dysregulation of cell death is a recognised characteristic of COPD lung tissue and is linked with the development of an emphysematous phenotype, as documented in several studies.^57^, ^58^, ^59^, ^60^ The pronounced activation of HIF-1α in ACO, but not in COPD, may drive the extensive airway remodelling seen in ACO, distinguishing it from the more severe emphysema and parenchymal destruction characteristic of COPD.

Although our study provides valuable insights into the characterisation of respiratory phenotypes, several limitations should be acknowledged. Smoking status was determined through self-report, which, despite being supported by spirometric parameters, can be susceptible to recall bias, social desirability bias, and intentional underreporting. Future studies could improve the accuracy of smoking status assessment by incorporating biochemical verification methods such as carbon monoxide monitoring or salivary cotinine measurements. Additionally, the COPDGene Study did not capture the specific nature of exacerbations, but further investigation into triggers such as infections (bacterial or viral) and environmental (air pollutants, allergens) exposures could offer deeper insights into the mechanisms driving exacerbations across disease phenotypes.

Another important limitation is the absence of detailed asthma endotyping, which would have enriched our understanding of asthma heterogeneity in smokers. Including specific asthma endotyping, such as distinguishing between T_H_2-high (eosinophilic) and T_H_2-low (neutrophilic), or paucigranulocytic subtypes, would have provided a clearer picture of the interaction between smoking and asthma inflammation. While we focused on systemic inflammatory markers through CBC analysis, this may not fully capture airway-specific inflammation, as peripheral blood eosinophil counts do not always correlate with airway eosinophilia.^61^ Considering that NETosis has also been linked to more severe asthma phenotypes, further investigation into the composition of asthma endotypes, particularly neutrophilic asthma, would provide valuable therapeutic insights.

Our findings contribute to the broader discourse on the classification of obstructive lung diseases, highlighting the complex interplay between clinical manifestations, functional deficits, systemic inflammation, immune mechanisms, and structural alterations. This holistic perspective paves the way for targeted therapies, improved diagnostics, and personalised treatments.

## Contributors

V.D.F. and S.T.W. conceptualised the study. V.D.F. analysed the data, generated figures, and wrote the first draft. A.S, P.J.C, and C.P.H. contributed to patient data acquisition and provided critical feedback. All authors contributed to discussions, provided critical feedback, edited, and approved the manuscript prior to submission.

## Data sharing statement

The COPDGene Study data is available from the NCBI database of Genotypes and Phenotypes (dbGaP), accessions phs000179 and phs000765.

## Code sharing

Code used for patient data analysis is available at https://github.com/vrushali-broad/COPDGene.git.

## Declaration of interests

S.T.W. receives royalties from UpToDate and serves on the Board of Histolix, a digital pathology company. C.P.H. reports research grants from Alpha-1 foundation, Bayer, Boehringer-Ingelheim, and Vertex, as well as consulting fees from Apogee therapeutics, Chiesi, Ono Pharma, Sanofi, and Takeda and Verona Pharma, unrelated to this manuscript. PJC reports grants from Sanofi and Bayer, as well as consulting fees from Verona Pharma and Genentech. The remaining authors declare no competing interests.

## Appendix A Supplementary data

Fig. S1 **Prevalence of emphysema and wheezing among different clinical phenotype groups**. Bar plots displaying the proportion of participants with **(a)** clinically diagnosed emphysema and **(b)** reported episodes of wheezing. Error bars represent 95% confidence intervals, reflecting the variability and precision of the estimates within each group. Statistical significance was assessed using the Chi-squared test with Bonferroni correction for multiple comparisons. Significance levels are indicated as: ns – not significant, ∗P≤0.05, ∗∗$P\leq 10^{-2}$, ∗∗∗$P\leq 10^{-3}$, ∗∗∗∗$P\leq 10^{-4}$.

Fig. S2 ***Model diagnostics for pulmonary function tests post*****Yeo-Johnson *transformation across respiratory conditions.*** Diagnostic plots for residuals of **(a)** FEV_1_/FVC ratio and **(b)** Resting SaO_2_. The left panels show histograms of residuals with associated Kolmogorov-Smirnov (KS) test p-values for normality. The middle panels display Q-Q plots, comparing sample quantiles to theoretical quantiles, assessing the normality of residuals. The right panels present residuals vs. fitted values with Breusch-Pagan test p-values, evaluating heteroscedasticity. HC3 robust standard errors were applied to correct for heteroscedasticity in the model estimates, but since this correction does not impact the appearance of the residuals vs. fitted values plots, the plots are shown in their original form.

Fig. S3 **Association of Disease Status with Pulmonary Function Adjusted for Confounding Factors in the COPDGene Cohort. (a)** Forest plot of adjusted estimates for FEV_1_/FVC, with each dot representing the estimate adjusted for age, sex, race, and height, and horizontal lines depicting 95% confidence intervals based on 1,000 bootstrap iterations. **(b)** Boxplot showing resting SaO_2_ percentages by disease category, with significance determined via one-way ANOVA followed by Tukey’s post-hoc test. **(c)** Forest plot of the adjusted estimates for resting SaO_2_, similarly adjusted for confounding factors. The control group serves as the reference for comparison with asthma, COPD, and ACO groups. P-values in the forest plots were derived from bootstrapped regression models and adjusted using the Benjamini-Hochberg (BH) correction. Statistical significance is marked (ns = not significant, ∗P≤0.05, ∗∗$P\leq 10^{-2}$, ∗∗∗$P\leq 10^{-3}$, ∗∗∗∗$P\leq 10^{-4}$), highlighting the correlations between disease categories and pulmonary function measures after controlling for potential confounders.

Fig. S4 **Interrelationship of Leukocyte Subtypes and Distribution of Blood Cell Counts by Clinical Phenotypes. (a)** Boxplot showing the distribution of lymphocyte counts (K/μL) stratified by disease categories – control, asthma, COPD, and ACO groups — displaying median values, interquartile ranges, and outliers. The sample size for each group is provided at the top of the plot. Statistical significance between groups was assessed using the Mann-Whitney U test with Bonferroni correction, indicated where applicable (ns = not significant, ∗∗∗$P\leq 10^{-3}$). Panels **(b-e)** display heatmaps representing correlation matrix and coefficients among different leukocyte subtypes, including WBC, eosinophils, neutrophils, monocytes, and lymphocytes in **(b)** Control, **(c)** Asthma, **(d)** COPD, **(e)** ACO clinical phenotypes, with colour intensity proportional to the correlation magnitude (blue for positive, red for negative).

Fig. S5 ***Differential NF-kappa B Signaling Pathway Activation in COPD and ACO derived from KEGG pathway analysis.*** This figure illustrates the NF-kappa B signaling pathway in **(a)** COPD and **(b)** ACO clinical phenotypes, with upregulated components marked in green and downregulated components in pink. These pathway maps highlight distinct and overlapping molecular signatures between COPD and ACO, revealing specific points of divergence that may inform targeted therapeutic strategies.

Fig. S6 **Neutrophil Extracellular Trap Formation Pathway in COPD Derived from KEGG Enrichment Analysis.** This pathway diagram depicts the molecular mechanisms involved in neutrophil extracellular trap (NET) formation in COPD. Upregulated components are marked in green, while downregulated components are highlighted in pink. The illustration reveals key elements of the NET formation process, including the activation of signalling pathways like MAPK and Toll-like receptor (TLR) signalling, leading to the release of NETs, which play a role in trapping and killing pathogens. This pathway also underscores the complex interactions between immune response, inflammation, and cell death mechanisms in COPD, providing insights into potential therapeutic targets.

Fig. S7 ***Model diagnostics for quantitative CT scan derived pulmonary metrics post quantile transformation across respiratory conditions.*** Diagnostic plots evaluating the assumptions of linear regression models for six pulmonary metrics, with estimates derived from 1,000 bootstrap iterations to ensure robustness. Metrics include (a) Emphysema (%), (b) Lung Density, (c) PRM^fSAD^, (d) Gas Trapping, (e) Pi10, and (f) Wall Area Thickness. The left panels show histograms of residuals with Kolmogorov-Smirnov (KS) test p-values, indicating residual normality. Middle panels feature Q-Q plots comparing residuals to a theoretical normal distribution, supporting normality. Right panels display residuals vs. fitted values with Breusch-Pagan test p-values assessing heteroscedasticity. Despite some heteroscedasticity indicated by the Breusch-Pagan test, HC3 robust standard errors were applied to correct for it. Overall, these diagnostics confirm that the models adequately meet assumptions of normality and homoscedasticity for analyzing CT-derived metrics.

Fig. S8 **Adjusted associations between disease status and CT-derived pulmonary metrics in the COPDGene cohort.** Forest plots **(****a-f****)** illustrate the adjusted effect estimates for **(a)** percentage of emphysema (< -950 HU), **(b)** lung density (Perc15), **(c)** Parametric Response Mapping of small airways disease (PRM^fSAD^), **(d)** gas trapping (< -856 HU), **(e)** Pi10, and **(f)** wall area percentage, with respect to the stratification of asthma, COPD, and ACO phenotypes. Each plot depicts the estimates, 95% confidence intervals, and p-values after adjusting for confounders such as age, BMI, gender, race, smoking status, and CT scanner model. Points represent the estimated effect size, with horizontal lines indicating the confidence intervals; points to the right (green) or left (orange) reflect positive or negative associations, respectively. Statistical significance was assessed using 1,000 bootstrap iterations, with p-values adjusted by the Benjamini-Hochberg (BH) correction. Statistically significant differences are indicated by asterisks, with p-values annotated alongside each estimate (ns = not significant, ∗P≤0.05, ∗∗$P\leq 10^{-2}$, ∗∗∗$P\leq 10^{-3}$, ∗∗∗∗$P\leq 10^{-4}$).
