# Supplementary figures and images for: Distinct physiological, transcriptomic, and imaging characteristics of asthma-COPD overlap compared to asthma and COPD in smokers
Source: eBioMedicine. 2024 Nov 23;110:105453. doi: 10.1016/j.ebiom.2024.105453 (PMC11621799; doi:10.1016/j.ebiom.2024.105453)

**a**  
Subjects with Emphysema (%)

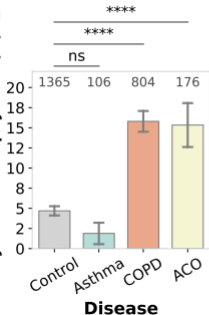

**b**  
Subjects with Wheeze (%)

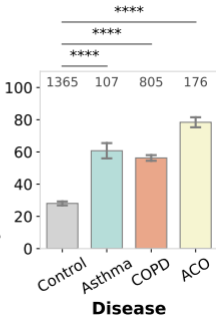

Supplement: Fig. S1 — Prevalence of emphysema and wheezing among different clinical phenotype groups. Bar plots displaying the proportion of participants with (a) clinically diagnosed emphysema and (b) reported episodes of wheezing. Error bars represent 95% confidence intervals, reflecting the variability and precision of the estimates within each group. Statistical significance was assessed using the Chi-squared test with Bonferroni correction for multiple comparisons. Significance levels are indicated as: ns – not significant, ∗P≤0.05, ∗∗P≤10−2, ∗∗∗P≤10−3, ∗∗∗∗P≤10−4. [file mmc1.pdf]

**a****FEV<sub>1</sub>/FVC**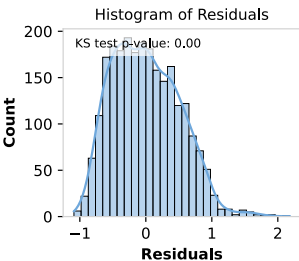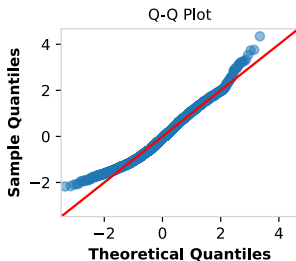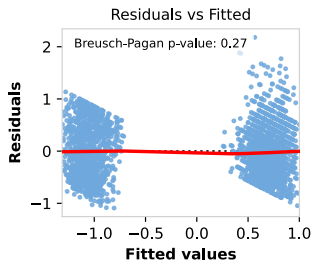**b****Resting SaO<sub>2</sub>**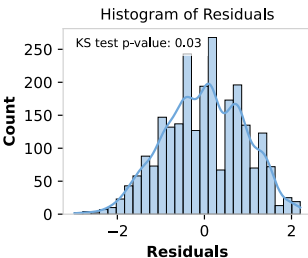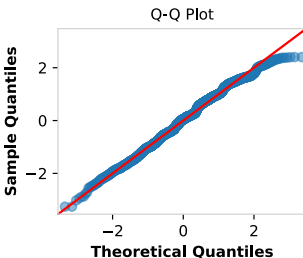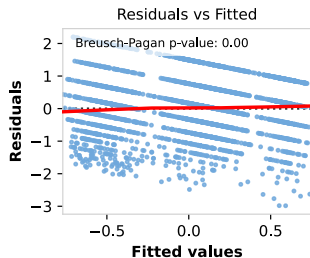

Supplement: Fig. S2 — Model diagnostics for pulmonary function tests postYeo-Johnson transformation across respiratory conditions. Diagnostic plots for residuals of (a) FEV1/FVC ratio and (b) Resting SaO2. The left panels show histograms of residuals with associated Kolmogorov-Smirnov (KS) test p-values for normality. The middle panels display Q-Q plots, comparing sample quantiles to theoretical quantiles, assessing the normality of residuals. The right panels present residuals vs. fitted values with Breusch-Pagan test p-values, evaluating heteroscedasticity. HC3 robust standard errors were applied to correct for heteroscedasticity in the model estimates, but since this correction does not impact the appearance of the residuals vs. fitted values plots, the plots are shown in their original form. [file mmc2.pdf]

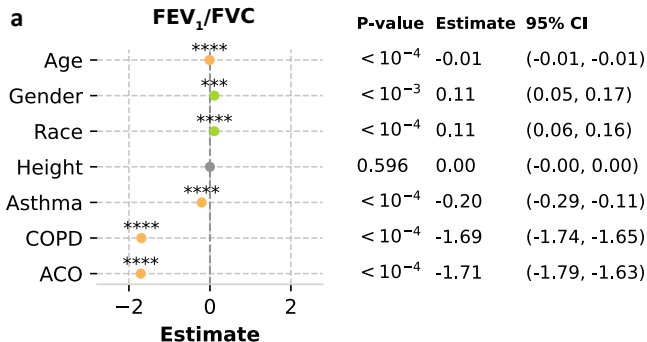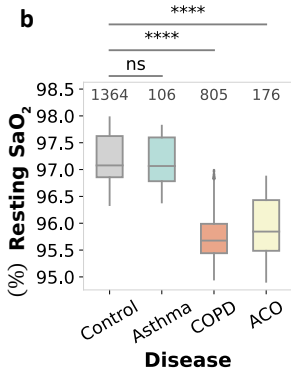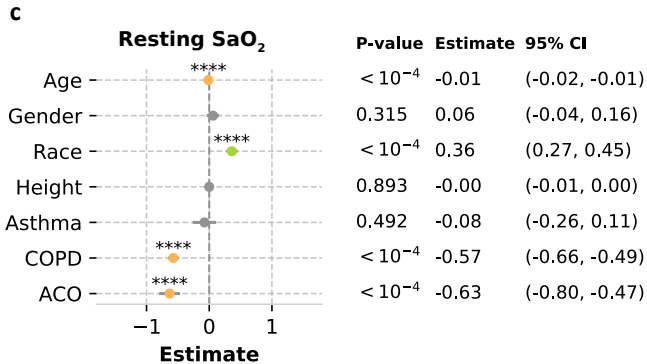

Supplement: Fig. S3 — Association of Disease Status with Pulmonary Function Adjusted for Confounding Factors in the COPDGene Cohort. (a) Forest plot of adjusted estimates for FEV1/FVC, with each dot representing the estimate adjusted for age, sex, race, and height, and horizontal lines depicting 95% confidence intervals based on 1,000 bootstrap iterations. (b) Boxplot showing resting SaO2 percentages by disease category, with significance determined via one-way ANOVA followed by Tukey’s post-hoc test. (c) Forest plot of the adjusted estimates for resting SaO2, similarly adjusted for confounding factors. The control group serves as the reference for comparison with asthma, COPD, and ACO groups. P-values in the forest plots were derived from bootstrapped regression models and adjusted using the Benjamini-Hochberg (BH) correction. Statistical significance is marked (ns = not significant, ∗P≤0.05, ∗∗P≤10−2, ∗∗∗P≤10−3, ∗∗∗∗P≤10−4), highlighting the correlations between disease categories and pulmonary function measures after controlling for potential confounders. [file mmc3.pdf]

# NEUTROPHIL EXTRACELLULAR TRAP INFORMATION

**Downregulated**  
**Upregulated**

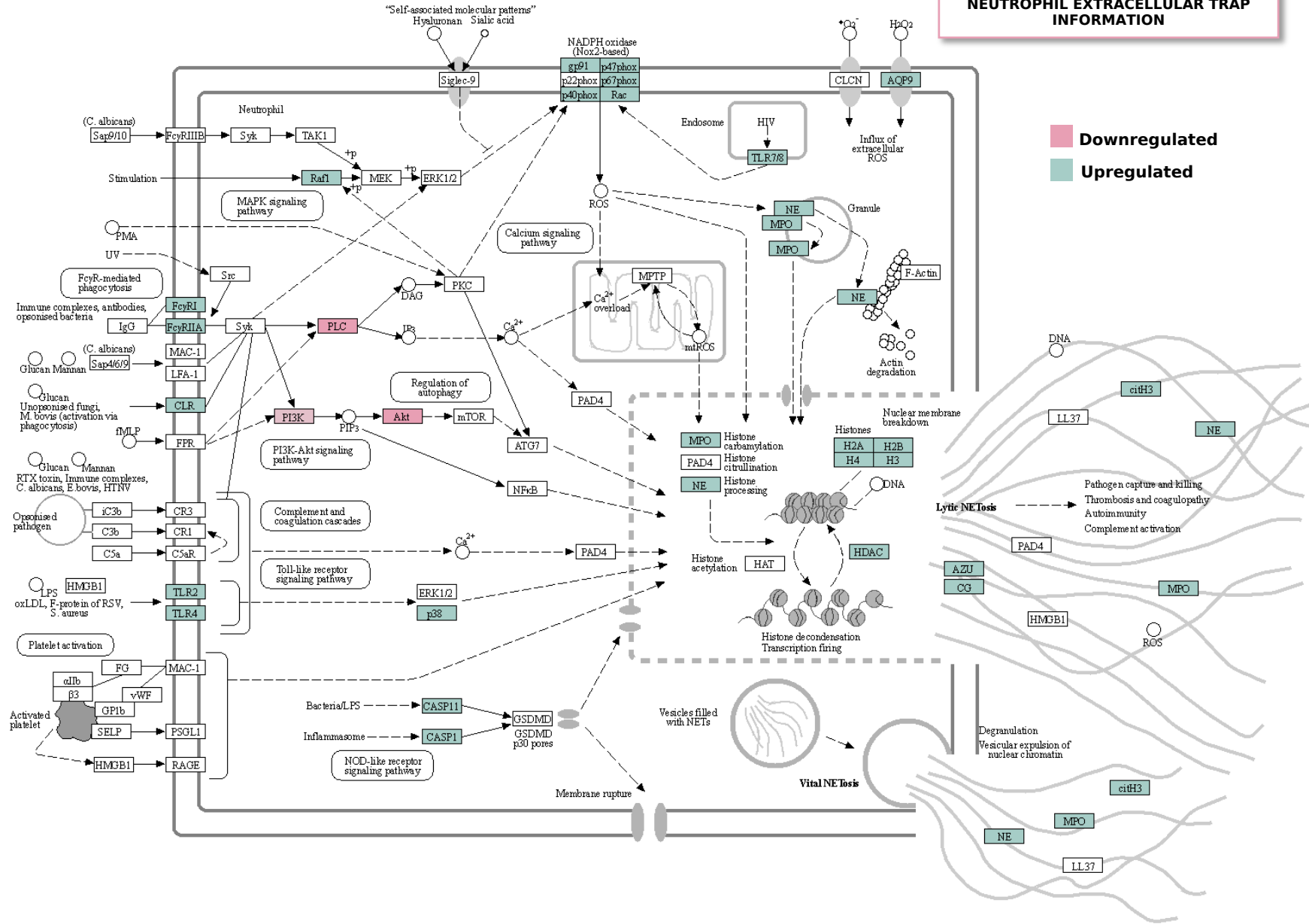

Supplement: Fig. S6 — Neutrophil Extracellular Trap Formation Pathway in COPD Derived from KEGG Enrichment Analysis. This pathway diagram depicts the molecular mechanisms involved in neutrophil extracellular trap (NET) formation in COPD. Upregulated components are marked in green, while downregulated components are highlighted in pink. The illustration reveals key elements of the NET formation process, including the activation of signalling pathways like MAPK and Toll-like receptor (TLR) signalling, leading to the release of NETs, which play a role in trapping and killing pathogens. This pathway also underscores the complex interactions between immune response, inflammation, and cell death mechanisms in COPD, providing insights into potential therapeutic targets. [file mmc6.pdf]

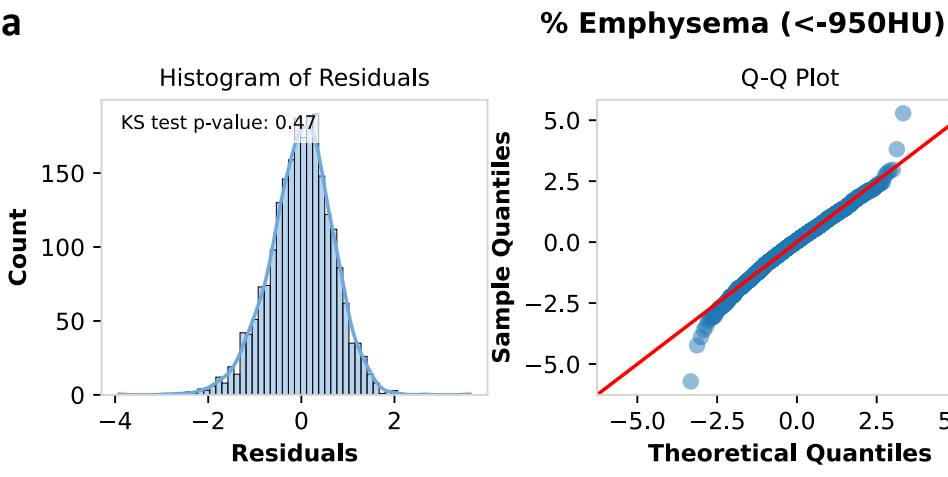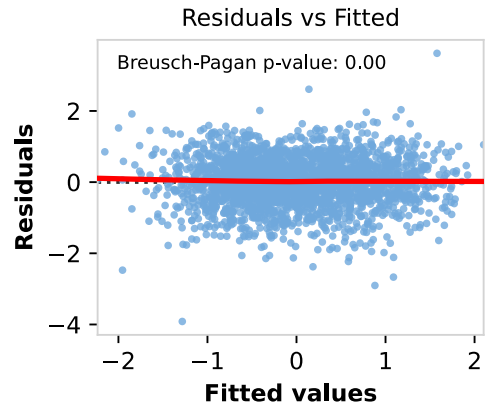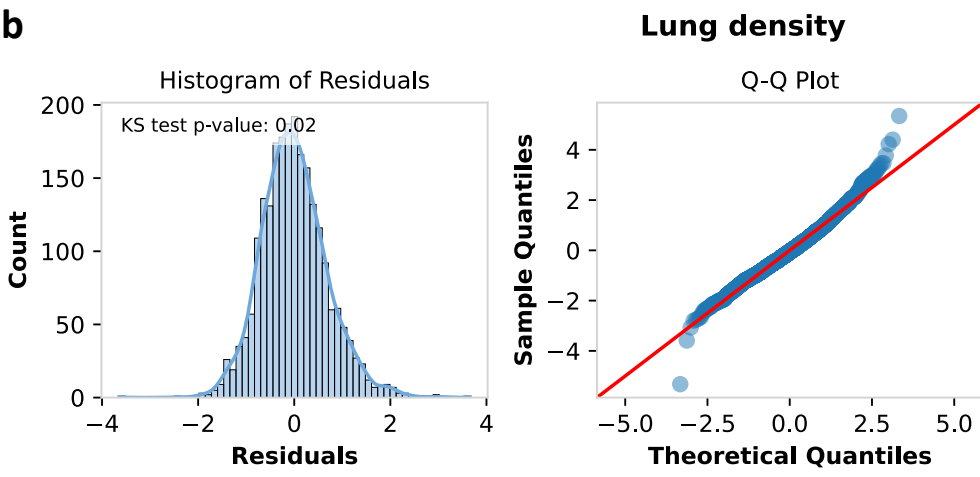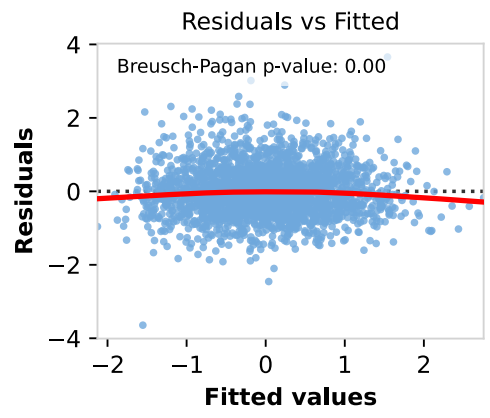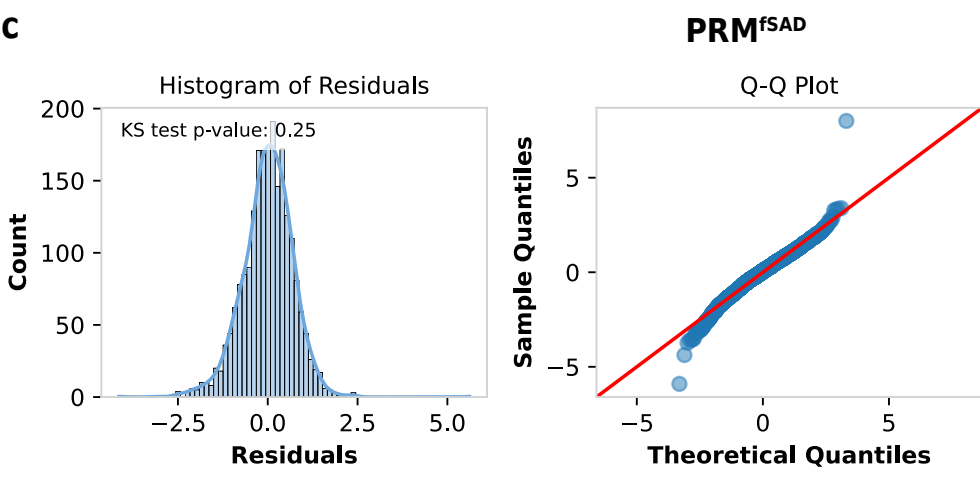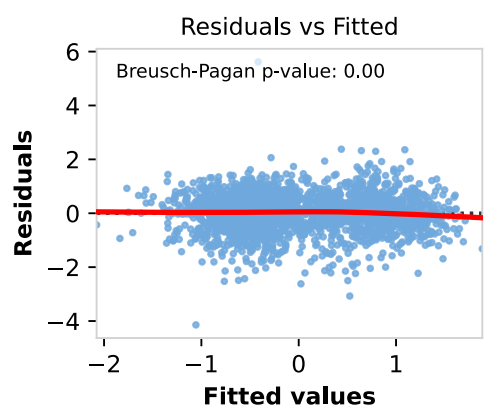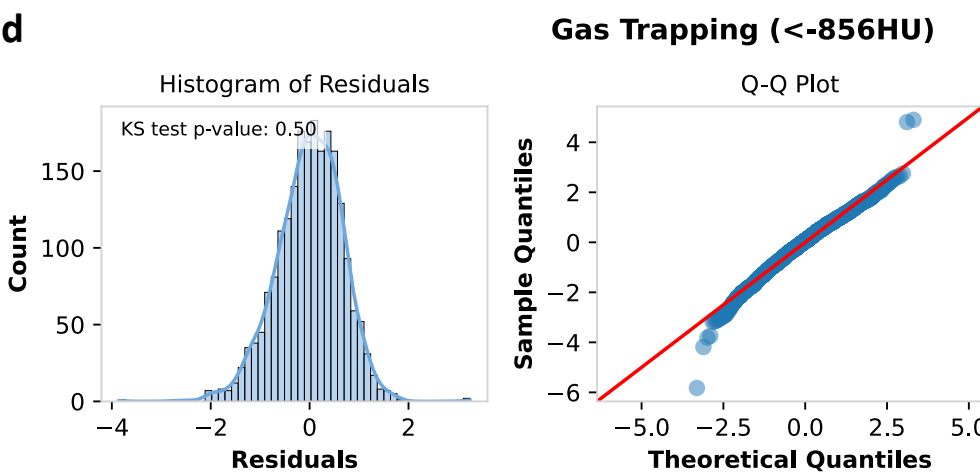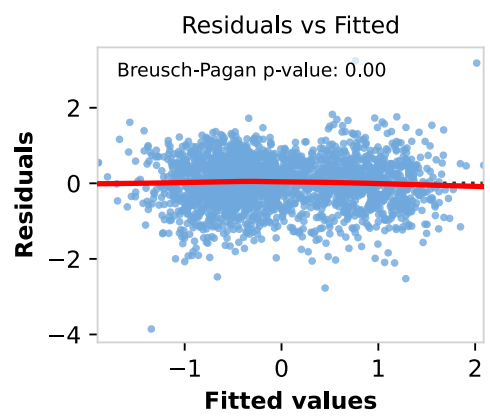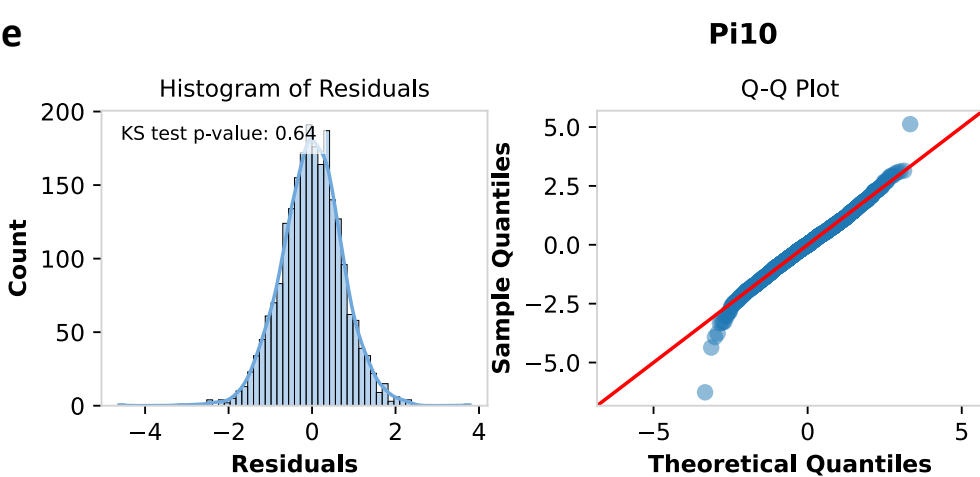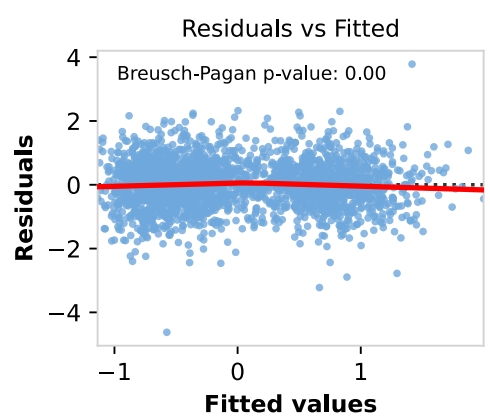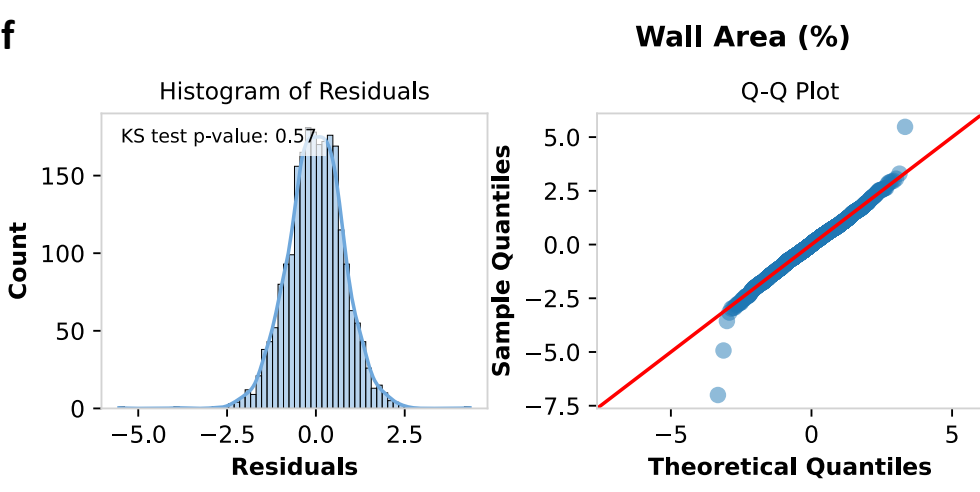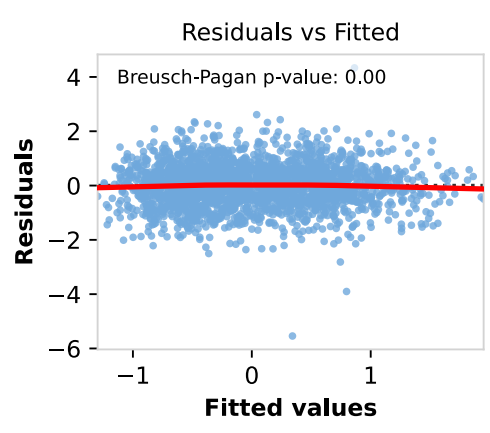

Supplement: Fig. S7 — Model diagnostics for quantitative CT scan derived pulmonary metrics post quantile transformation across respiratory conditions. Diagnostic plots evaluating the assumptions of linear regression models for six pulmonary metrics, with estimates derived from 1,000 bootstrap iterations to ensure robustness. Metrics include (a) Emphysema (%), (b) Lung Density, (c) PRMfSAD, (d) Gas Trapping, (e) Pi10, and (f) Wall Area Thickness. The left panels show histograms of residuals with Kolmogorov-Smirnov (KS) test p-values, indicating residual normality. Middle panels feature Q-Q plots comparing residuals to a theoretical normal distribution, supporting normality. Right panels display residuals vs. fitted values with Breusch-Pagan test p-values assessing heteroscedasticity. Despite some heteroscedasticity indicated by the Breusch-Pagan test, HC3 robust standard errors were applied to correct for it. Overall, these diagnostics confirm that the models adequately meet assumptions of normality and homoscedasticity for analyzing CT-derived metrics. [file mmc7.pdf]

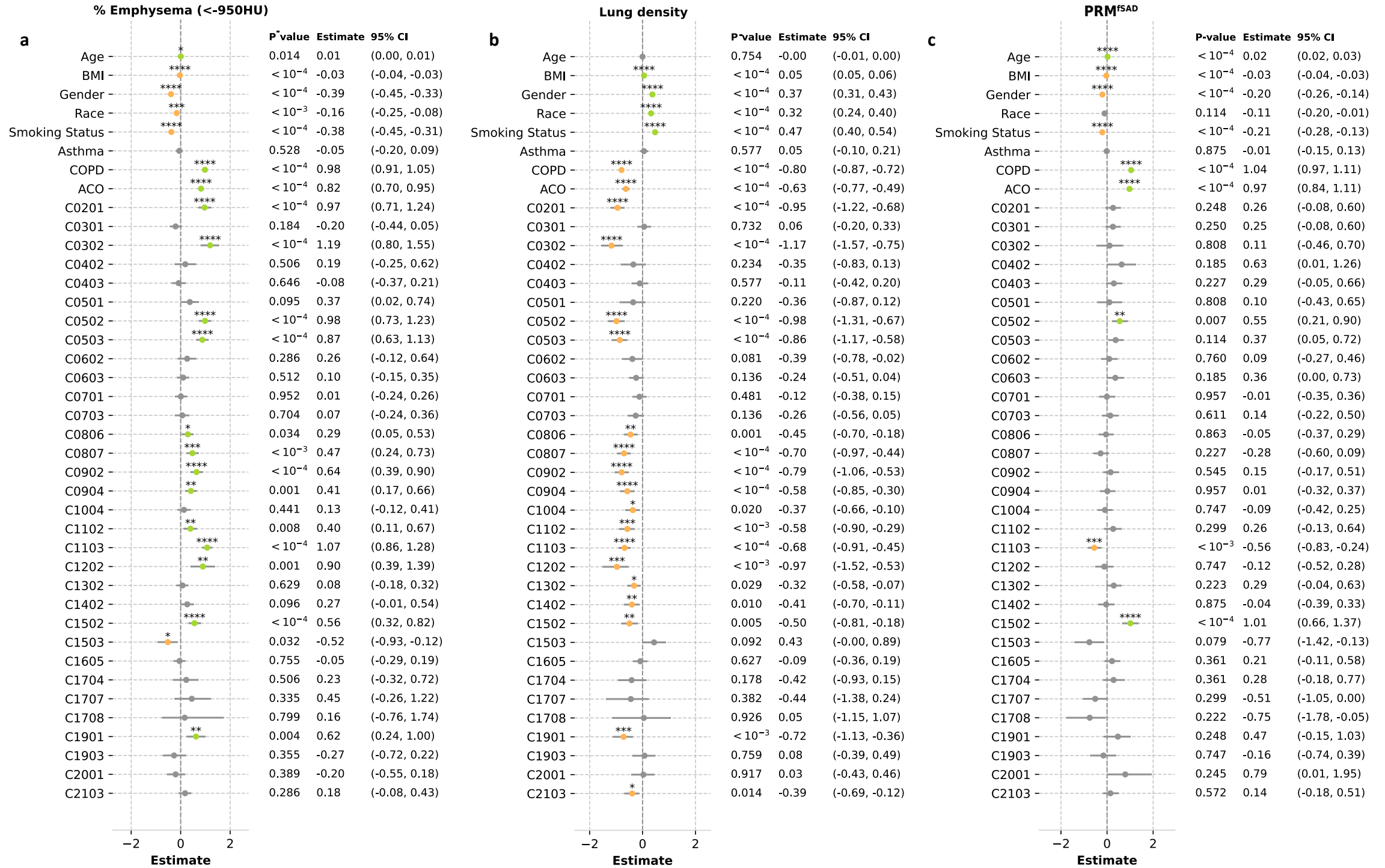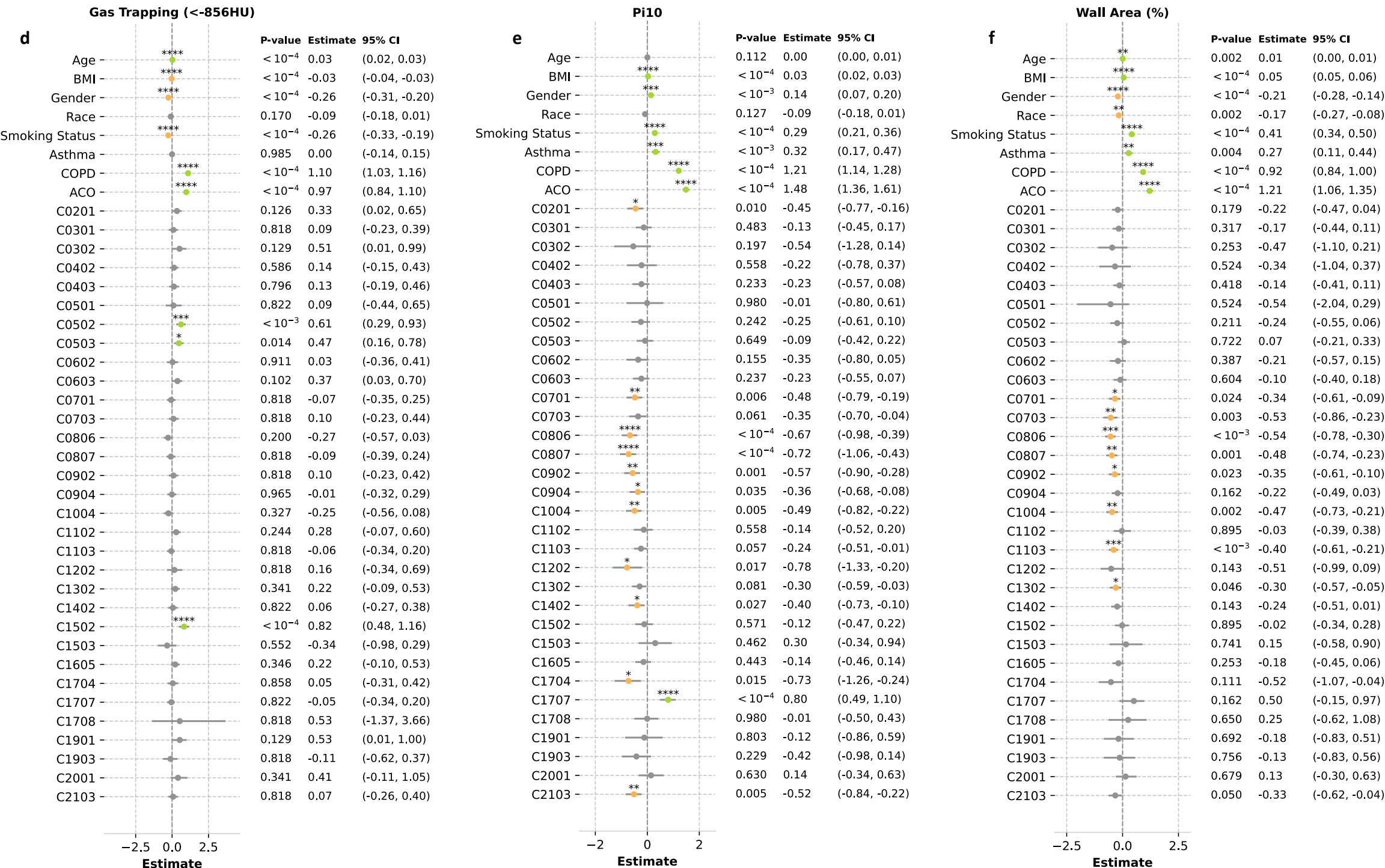

Supplement: Fig. S8 — Adjusted associations between disease status and CT-derived pulmonary metrics in the COPDGene cohort. Forest plots (a-f) illustrate the adjusted effect estimates for (a) percentage of emphysema (< -950 HU), (b) lung density (Perc15), (c) Parametric Response Mapping of small airways disease (PRMfSAD), (d) gas trapping (< -856 HU), (e) Pi10, and (f) wall area percentage, with respect to the stratification of asthma, COPD, and ACO phenotypes. Each plot depicts the estimates, 95% confidence intervals, and p-values after adjusting for confounders such as age, BMI, gender, race, smoking status, and CT scanner model. Points represent the estimated effect size, with horizontal lines indicating the confidence intervals; points to the right (green) or left (orange) reflect positive or negative associations, respectively. Statistical significance was assessed using 1,000 bootstrap iterations, with p-values adjusted by the Benjamini-Hochberg (BH) correction. Statistically significant differences are indicated by asterisks, with p-values annotated alongside each estimate (ns = not significant, ∗P≤0.05, ∗∗P≤10−2, ∗∗∗P≤10−3, ∗∗∗∗P≤10−4). [file mmc8.pdf]
