# Supplementary material for: Distinct physiological, transcriptomic, and imaging characteristics of asthma-COPD overlap compared to asthma and COPD in smokers
Source: eBioMedicine. 2024 Nov 23;110:105453. doi: 10.1016/j.ebiom.2024.105453 (PMC11621799; doi:10.1016/j.ebiom.2024.105453)
Supplement: Fig. S4 — Interrelationship of Leukocyte Subtypes and Distribution of Blood Cell Counts by Clinical Phenotypes. (a) Boxplot showing the distribution of lymphocyte counts (K/μL) stratified by disease categories – control, asthma, COPD, and ACO groups — displaying median values, interquartile ranges, and outliers. The sample size for each group is provided at the top of the plot. Statistical significance between groups was assessed using the Mann-Whitney U test with Bonferroni correction, indicated where applicable (ns = not significant, ∗∗∗P≤10−3). Panels (b-e) display heatmaps representing correlation matrix and coefficients among different leukocyte subtypes, including WBC, eosinophils, neutrophils, monocytes, and lymphocytes in (b) Control, (c) Asthma, (d) COPD, (e) ACO clinical phenotypes, with colour intensity proportional to the correlation magnitude (blue for positive, red for negative). [file mmc4.pdf]

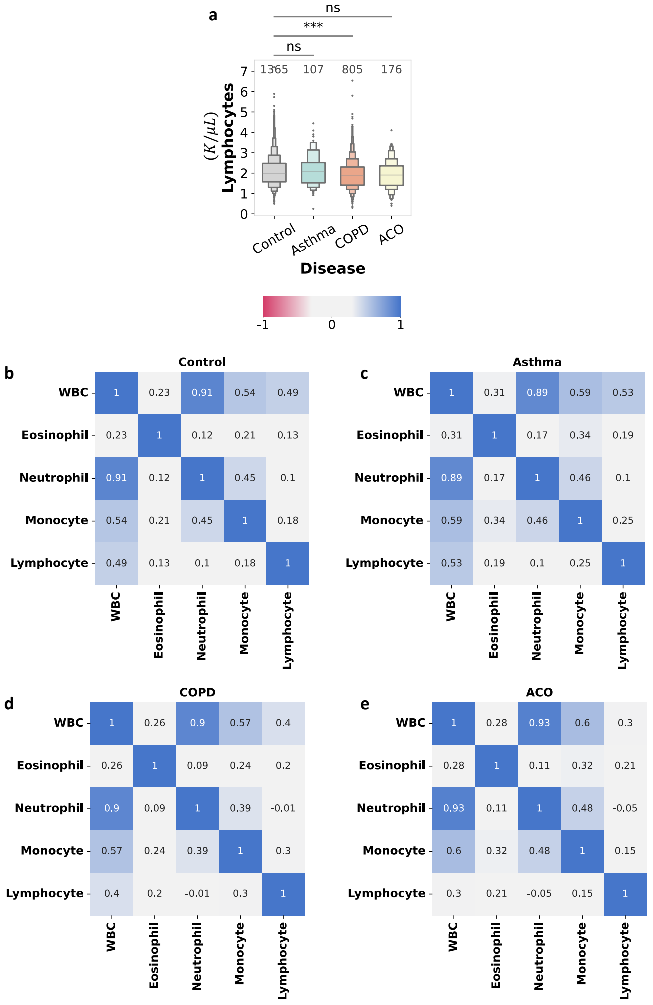
a
ns
***
ns
1365
107
805
176
Lymphocytes
(K/μL)
0
1
2
3
4
5
6
7
Control
Asthma
COPD
ACO
Disease
-1
0
1
b
Control
WBC
Eosinophil
Neutrophil
Monocyte
Lymphocyte
1 0.23 0.91 0.54 0.49
0.23 1 0.12 0.21 0.13
0.91 0.12 1 0.45 0.1
0.54 0.21 0.45 1 0.18
0.49 0.13 0.1 0.18 1
c
Asthma
1 0.31 0.89 0.59 0.53
0.31 1 0.17 0.34 0.19
0.89 0.17 1 0.46 0.1
0.59 0.34 0.46 1 0.25
0.53 0.19 0.1 0.25 1
d
COPD
1 0.26 0.9 0.57 0.4
0.26 1 0.09 0.24 0.2
0.9 0.09 1 0.39 -0.01
0.57 0.24 0.39 1 0.3
0.4 0.2 -0.01 0.3 1
e
ACO
1 0.28 0.93 0.6 0.3
0.28 1 0.11 0.32 0.21
0.93 0.11 1 0.48 -0.05
0.6 0.32 0.48 1 0.15
0.3 0.21 -0.05 0.15 1
