# Supplementary material for: Distinct physiological, transcriptomic, and imaging characteristics of asthma-COPD overlap compared to asthma and COPD in smokers
Source: eBioMedicine. 2024 Nov 23;110:105453. doi: 10.1016/j.ebiom.2024.105453 (PMC11621799; doi:10.1016/j.ebiom.2024.105453)

NF-KAPPA B SIGNALING PATHWAY

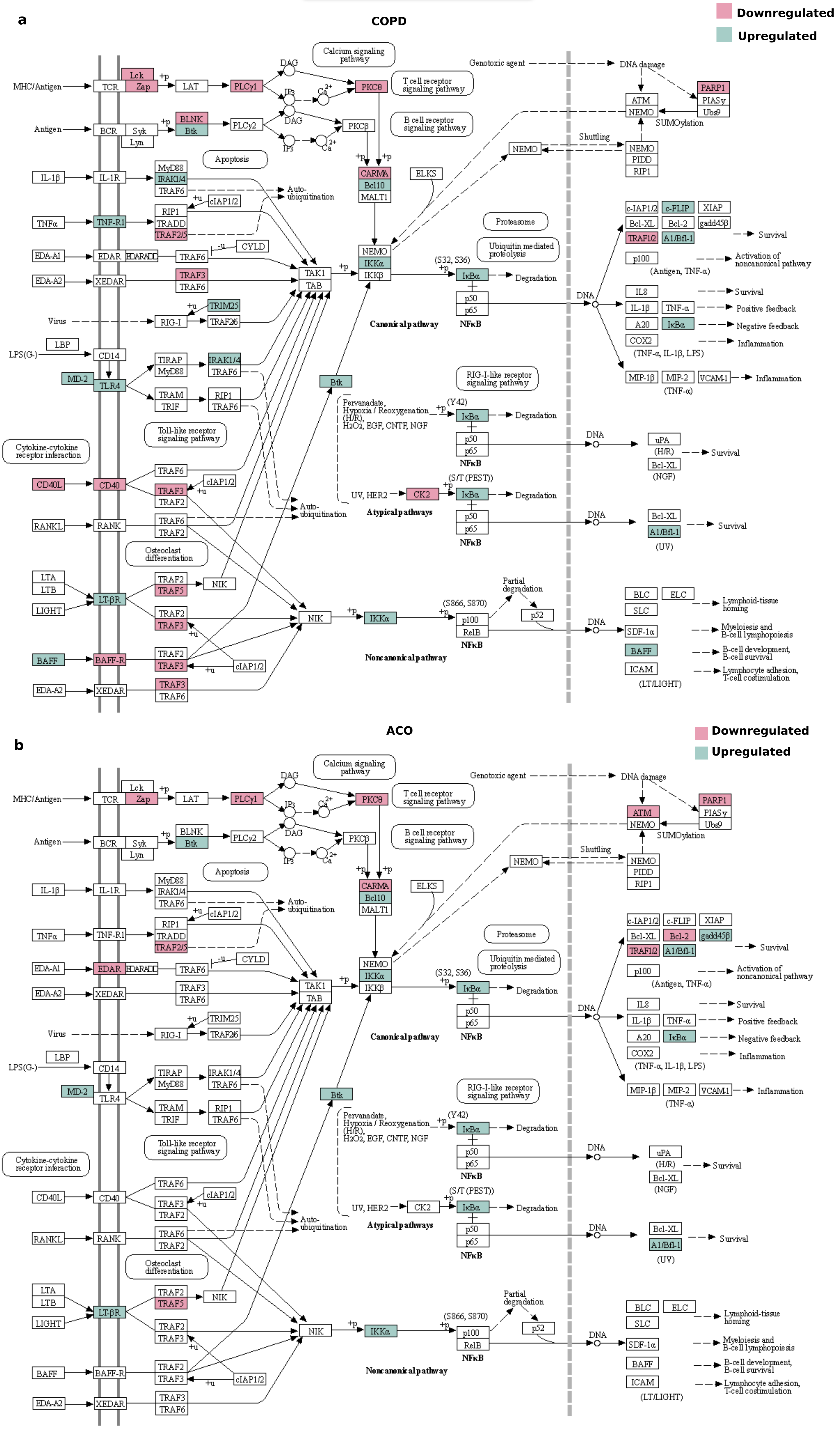

Supplement: Fig. S5 — Differential NF-kappa B Signaling Pathway Activation in COPD and ACO derived from KEGG pathway analysis. This figure illustrates the NF-kappa B signaling pathway in (a) COPD and (b) ACO clinical phenotypes, with upregulated components marked in green and downregulated components in pink. These pathway maps highlight distinct and overlapping molecular signatures between COPD and ACO, revealing specific points of divergence that may inform targeted therapeutic strategies. [file mmc5.pdf]
